# An integrated meta-analysis of peripheral blood metabolites and biological functions in major depressive disorder

**DOI:** 10.1038/s41380-020-0645-4

**Published:** 2020-01-20

**Authors:** Juncai Pu, Yiyun Liu, Hanping Zhang, Lu Tian, Siwen Gui, Yue Yu, Xiang Chen, Yue Chen, Lining Yang, Yanqin Ran, Xiaogang Zhong, Shaohua Xu, Xuemian Song, Lanxiang Liu, Peng Zheng, Haiyang Wang, Peng Xie

**Affiliations:** 1 NHC Key Laboratory of Diagnosis and Treatment on Brain Functional Diseases, The First Affiliated Hospital of Chongqing Medical University, Yuzhong District, Chongqing, 400016 China; 2 Department of Neurology, The First Affiliated Hospital of Chongqing Medical University, Chongqing, 400016 China; 3 Chongqing Key Laboratory of Neurobiology, Chongqing, 400016 China; 4 College of Medical Informatics, Chongqing Medical University, Chongqing, 400016 China; 5 Department of Health Sciences Research, Mayo Clinic, Rochester, MN 55901 USA

**Keywords:** Biochemistry, Diagnostic markers, Depression

## Abstract

Major depressive disorder (MDD) is a serious mental illness, characterized by high morbidity, which has increased in recent decades. However, the molecular mechanisms underlying MDD remain unclear. Previous studies have identified altered metabolic profiles in peripheral tissues associated with MDD. Using curated metabolic characterization data from a large sample of MDD patients, we meta-analyzed the results of metabolites in peripheral blood. Pathway and network analyses were then performed to elucidate the biological themes within these altered metabolites. We identified 23 differentially expressed metabolites between MDD patients and controls from 46 studies. MDD patients were characterized by higher levels of asymmetric dimethylarginine, tyramine, 2-hydroxybutyric acid, phosphatidylcholine (32:1), and taurochenodesoxycholic acid and lower levels of l-acetylcarnitine, creatinine, l-asparagine, l-glutamine, linoleic acid, pyruvic acid, palmitoleic acid, l-serine, oleic acid, myo-inositol, dodecanoic acid, l-methionine, hypoxanthine, palmitic acid, l-tryptophan, kynurenic acid, taurine, and 25-hydroxyvitamin D compared with controls. l-tryptophan and kynurenic acid were consistently downregulated in MDD patients, regardless of antidepressant exposure. Depression rating scores were negatively associated with decreased levels of l-tryptophan. Pathway and network analyses revealed altered amino acid metabolism and lipid metabolism, especially for the tryptophan–kynurenine pathway and fatty acid metabolism, in the peripheral system of MDD patients. Taken together, our integrated results revealed that metabolic changes in the peripheral blood were associated with MDD, particularly decreased l-tryptophan and kynurenic acid levels, and alterations in the tryptophan–kynurenine and fatty acid metabolism pathways. Our findings may facilitate biomarker development and the elucidation of the molecular mechanisms that underly MDD.

## Introduction

Major depressive disorder (MDD) is a mental disorder with symptoms that include low mood for at least 2 weeks, loss of interest, fatigue, and feelings of guilt [1]. MDD is a serious mental illness, characterized by high morbidity and a high suicide rate [2], and was the leading cause of disability in 2016 [3]. The lifetime prevalence of MDD has been reported to be ~20%, with increasing morbidity during recent decades [4, 5]. However, the clinical diagnosis of MDD remains underestimated because of high diagnostic error rates in primary care [6]. As a complex and heterogeneous mental disease, no robust peripheral biomarkers currently exist for MDD, and the molecular mechanisms underlying this disease remain unclear, which has impeded objective diagnoses and clinical therapy [7, 8].

Among the various omic techniques, metabolomics, which can be used to characterize the metabolic profiles of biological samples, is the best tool for determining phenotypes and can be utilized to identify disease-specific biomarkers and mechanisms [9, 10]. Mass spectrometry (MS)- and nuclear magnetic resonance (NMR)-based techniques have been widely used during biomarker development and to examine the molecular mechanisms that underly neuropsychiatric diseases [11, 12]. A range of studies has revealed perturbed metabolomes in the peripheral tissues of MDD patients. For example, we previously reported the use of plasma metabolic profiles as potential biomarkers among adult and adolescent patients with MDD [13, 14]. Further, we found that the dysbiosis of the gut microbiome may play a causal role in the induction of depressive-like behaviors in rodents via effects on metabolism [15]. Other studies have also reported that metabolic changes in the periphery may represent potential therapeutic targets for depression [16, 17]. These findings imply that metabolites may play important roles during the brain–body interactions involved in depression, and peripheral metabolite levels have been associated with hippocampal subfield volumes [18], hypothalamic pituitary adrenal axis activity [19], and neurocognitive function [20].

Despite advances in metabolomics research examining MDD, the majority of these nontargeted or targeted metabolomic studies have had small sample sizes and have reported inconsistent findings, limiting their clinical applicability. To date, a range of meta-analyses has reported decreased levels of tryptophan, kynurenic acid, and kynurenine and increased glutamate levels, in MDD patients [21–23]. Despite these studies, which have examined the levels of one or more metabolites, no meta-analyses have been performed examining the metabolomic profiles of MDD. A comprehensive bioinformatics analysis, based on the differential metabolites identified at the metabolomics level, may provide important insights into the pathological molecular mechanisms underlying MDD.

Thus, the aim of the present study was to identify metabolic changes in the peripheral blood of MDD patients. We first generated a curated list of metabolites in the peripheral blood of MDD patients, according to the results of preexisting studies, and performed a meta-analysis to identify differential metabolites, which may serve as robust biomarkers for the clinical diagnosis of MDD. We then performed comprehensive pathway and network analyses to examine the biological themes associated with these metabolic changes.

## Materials and methods

### Identification of relevant studies

A flowchart describing the process used to identify relevant studies is shown in Supplementary Fig. 1. Detailed methods describing the identification of relevant studies are provided in Supplementary Materials. In brief, clinical studies that compared metabolic changes between MDD and control groups were identified in MENDA (http://menda.cqmu.edu.cn:8080/index.php), our online database of existing metabolic characterization studies associated with depression. We updated our search up to January 2019, using our previously reported search terms [24]. In this study, we narrowed our selection according to the following steps. We included studies that compared metabolite levels in serum or plasma samples between MDD patients and controls and detected these changes using MS-based or NMR-based techniques.

### Data curation

We extracted study information and metabolite data from the selected studies. Recorded study information included methodological and demographic information, including the biological samples used, the recruiting area, sample size, mean age, percent of females, antidepressant-free patients, disease severity measure, and the analytical platform used. Curated metabolite data included each metabolite examined, with accompanying statistic data (mean, standard deviation, and sample size). Other data, such as standard errors, *p* values, or interquartile ranges, were transformed, as previously reported [25].

### Identification of differential metabolites in MDD

Meta-analyses were performed using statistical software (Stata v14.0; Stata Co., College Station, TX, USA), as follows. Only metabolites reported in at least three different datasets were selected for analysis [26]. Standardized effect sizes across studies were combined, based on the statistics reported for each metabolite (mean, standard deviation, and sample size) by the original reports. The standardized mean difference (SMD) and the 95% confidence interval (CI) were estimated. A random-effects model was used to determine the expected high degree of heterogeneity across studies, as reported in previous molecular studies [27, 28]. Positive and negative SMD values indicated higher and lower levels of metabolites in the MDD group, relative to the control group, respectively. Statistical significance was set at *p* < 0.05.

Publication bias was assessed using the Egger’s test, with a *p* value < 0.10 indicating the presence of publication bias [29]. The Duval and Tweedie’s trim and fill method was also used to reduce bias among the pooled estimates [30]. To test the sources of potential heterogeneity, subgroup analyses were performed, according to antidepressant use (yes or no) and the biological sample used (plasma or serum). Sensitivity analyses were performed for studies that recruited patients with a mean age of >18 years, by excluding studies that recruited pediatric patients, and for studies using MS analytic platforms, by excluding NMR studies. Meta-regression analyses were performed to investigate the influence of each variable (sample size, the proportion of females, mean age, and disease severity) on the SMD. Details describing disease severity assessment are provided in Supplementary Materials. Meta-regression analyses were only conducted for metabolites that were reported in at least ten datasets, as results using fewer datasets can lead to increased risk of spurious findings [31].

### Bioinformatics analysis

To reveal the biological functions of the differential metabolites and their interactions, we performed pathway and network analyses. Detailed methods are available in Supplementary Materials. In brief, altered metabolic pathways were identified, using metabolic pathway analysis in MetaboAnalyst 4.0 [32], which performed an enrichment analysis based on predefined KEGG pathways and differential metabolites [33]. Ingenuity pathways analysis (IPA, http://www.ingenuity.com) was used for further analysis. Canonical pathway analysis was performed to identify altered pathways within the Ingenuity Pathway Knowledge Base. For both metabolic pathway analysis and canonical pathway analysis, a pathway with a *p* value < 0.05 was considered to be significantly enriched. Network analysis was also performed in IPA to construct molecular networks, based on interactions among input metabolites and other biological molecules.

## Results

### Characteristics of the included studies

Lists of included and excluded studies are provided in Supplementary Data. A total of 49 comparisons (48 two-arm comparisons and 1 three-arm comparison) were included. The characteristics of the included studies are summarized in Supplementary Table 1. Of these comparisons, 47 used the MS platform, 33 measured metabolites in plasma, and 24 recruited antidepressant-free patients. All included studies examined the associations between metabolites and MDD using cross-sectional data. The sample sizes ranged from 16 to 2812 participants per study, with a median study size of 89 participants. The mean age of participants ranged from 14.0 to 72.2 years, with a median age of 37.1 years. The median proportion of females was 58.1%.

### Differential blood metabolites associated with MDD

A total of 83 metabolites, which were reported in at least three different datasets, were used for the differential metabolite identification (Table 1). A total of 23 metabolites were found to be significantly differentially regulated between the MDD and control groups (Supplementary Fig. 2). MDD patients were characterized by higher levels of asymmetric dimethylarginine, tyramine, 2-hydroxybutyric acid, phosphatidylcholine (32:1), and taurochenodesoxycholic acid and lower levels of l-acetylcarnitine, creatinine, l-asparagine, l-glutamine, linoleic acid, pyruvic acid, palmitoleic acid, l-serine, oleic acid, myo-inositol, dodecanoic acid, l-methionine, hypoxanthine, palmitic acid, l-tryptophan, kynurenic acid, taurine, and 25-hydroxyvitamin D. Forest plots for these differential metabolites are shown in Supplementary Fig. 3. The heterogeneity among effect sizes was significant for 15 differential metabolites (*p* for heterogeneity <0.05). The Egger test indicated potential publication bias for l-tryptophan (*p* = 0.004), l-serine (*p* = 0.034), 25-hydroxyvitamin D (*p* = 0.060), l-glutamine (*p* = 0.067), l-asparagine (*p* = 0.079), and tyramine (*p* = 0.091). After quantifying the potential effects of small-study bias, using the trim and fill method, the imputations resulted in larger summary SMDs (−0.70 for l-tryptophan; −1.80 for l-serine; −0.30 for 25-hydroxyvitamin D; −2.63 for l-glutamine; −2.20 for l-asparagine; and 1.12 for tyramine).

**Table 1:** Effect sizes of blood metabolites between patients with major depressive disorder and controls.

Metabolites	*N* Comparisons	*N* MDD/CON	SMD (95% CI)	*p* value^a^ (overall)	*I*^2^	*p* value^b^ (heterogeneity)	*p* value^c^ (Egger)
Kynurenic acid	13	797/653	−0.42 (−0.52 to −0.31)	<0.001	1.2%	0.434	0.647
2-Hydroxybutyric acid	3	76/83	1.02 (0.69 to 1.36)	<0.001	0.0%	0.652	0.990
Dodecanoic acid	4	151/114	−0.73 (−0.99 to −0.48)	<0.001	0.0%	0.544	0.495
25-Hydroxyvitamin D	3	1130/521	−0.29 (−0.39 to −0.18)	<0.001	0.0%	0.811	0.060
Tyramine	3	133/121	1.12 (0.70 to 1.55)	<0.001	58.8%	0.089	0.091
Phosphatidylcholine (32:1)	3	150/130	0.56 (0.32 to 0.80)	<0.001	0.0%	0.482	0.117
l-Tryptophan	26	2390/2928	−0.46 (−0.66 to −0.26)	<0.001	87.8%	<0.001	0.004
Myo-inositol	3	113/77	−0.79 (−1.17 to −0.41)	<0.001	28.3%	0.248	0.619
Linoleic acid	5	148/104	−0.99 (−1.46 to −0.51)	<0.001	63.4%	0.027	0.348
Oleic acid	5	148/104	−0.84 (−1.31 to −0.36)	0.001	63.6%	0.027	0.368
Taurochenodesoxycholic acid	4	176/180	0.33 (0.11 to 0.54)	0.003	0.0%	0.446	0.320
Creatinine	5	186/175	−1.79 (−3.02 to −0.56)	0.004	95.7%	<0.001	0.321
l-Glutamine	8	329/287	−1.28 (−2.19 to −0.37)	0.006	95.8%	<0.001	0.067
Asymmetric dimethylarginine	4	191/342	2.52 (0.68 to 4.36)	0.007	97.9%	<0.001	0.202
l-Acetylcarnitine	3	138/109	−2.06 (−3.58 to −0.54)	0.008	95.4%	<0.001	0.106
Hypoxanthine	6	259/386	−0.70 (−1.21 to −0.18)	0.009	85.9%	<0.001	0.391
Palmitic acid	4	139/94	−0.69 (−1.23 to −0.16)	0.011	71.2%	0.015	0.288
Pyruvic acid	3	101/92	−0.92 (−1.68 to −0.17)	0.017	80.7%	0.006	0.370
l-Asparagine	6	221/195	−1.33 (−2.45 to −0.21)	0.020	95.7%	<0.001	0.079
Palmitoleic acid	3	93/69	−0.90 (−1.70 to −0.10)	0.027	79.8%	0.007	0.228
l-Methionine	8	356/306	−0.73 (−1.40 to −0.06)	0.032	93.4%	<0.001	0.299
l-Serine	8	296/245	−0.90 (−1.73 to −0.07)	0.034	94.5%	<0.001	0.034
Taurine	6	270/218	−0.33 (−0.65 to −0.01)	0.043	65.0%	0.014	0.399
Glycine	8	325/262	−0.91 (−1.82 to 0.01)	0.051	95.7%	<0.001	0.096
Capric acid	4	151/114	−1.31 (−2.65 to 0.03)	0.056	95.6%	<0.001	0.033
l-Lysine	6	221/195	−1.03 (−2.10 to 0.04)	0.059	95.4%	<0.001	0.053
Citric acid	5	143/135	−0.96 (−1.97 to 0.05)	0.063	92.5%	<0.001	0.571
l-Valine	8	325/262	−0.71 (−1.48 to 0.06)	0.071	94.3%	<0.001	0.049
l-Kynurenine	18	2020/2595	−0.17 (−0.36 to 0.02)	0.077	81.9%	<0.001	0.948
l-Phenylalanine	7	271/245	0.68 (−0.08 to 1.44)	0.079	93.4%	<0.001	0.133
l-Threonine	8	296/245	−0.64 (−1.36 to 0.09)	0.085	93.1%	<0.001	0.088
Urea	3	113/89	−2.11 (−4.59 to 0.38)	0.097	97.9%	<0.001	0.018
Gluconic acid	3	89/75	−0.27 (−0.58 to 0.05)	0.099	0.0%	0.793	0.412
l-Tyrosine	8	329/287	−0.31 (−0.69 to 0.06)	0.099	79.4%	<0.001	0.954
Aminoadipic acid	5	190/136	1.15 (−0.22 to 2.51)	0.100	96.4%	<0.001	0.032
Gamma-aminobutyric acid	7	246/184	0.81 (−0.17 to 1.79)	0.104	94.5%	<0.001	0.775
Creatine	5	186/175	−0.72 (−1.61 to 0.16)	0.108	93.2%	<0.001	0.385
l-Alanine	8	325/262	−0.67 (−1.48 to 0.15)	0.109	94.8%	<0.001	0.060
Phosphatidylethanolamine (34:2)	3	150/130	0.38 (−0.12 to 0.87)	0.136	73.0%	0.025	0.146
Stearic acid	3	64/54	−0.42 (−1.00 to 0.16)	0.152	47.7%	0.148	0.938
l-Isoleucine	7	279/237	−0.58 (−1.40 to 0.25)	0.171	94.4%	<0.001	0.113
Succinic acid	4	163/139	0.69 (−0.35 to 1.74)	0.194	94.4%	<0.001	0.001
Ornithine	8	370/498	−0.31 (−0.78 to 0.17)	0.205	89.1%	<0.001	0.236
Homovanillic acid	3	117/114	1.36 (−0.80 to 3.52)	0.216	97.8%	<0.001	0.316
Glyceric acid	4	122/99	1.29 (−0.76 to 3.35)	0.217	97.1%	<0.001	0.613
l-Leucine	8	325/262	−0.14 (−0.37 to 0.08)	0.217	41.5%	0.102	0.870
Adenosine diphosphate	3	76/83	2.01 (−1.20 to 5.22)	0.220	98.0%	<0.001	0.448
l-Malic acid	3	76/83	0.87 (−0.57 to 2.31)	0.235	93.7%	<0.001	0.908
Indoleacetic acid	3	82/83	1.49 (−1.03 to 4.00)	0.247	97.6%	<0.001	0.300
Symmetric dimethylarginine	3	166/317	1.05 (−0.75 to 2.84)	0.253	97.7%	<0.001	0.384
Arachidonic acid	3	93/69	−0.52 (−1.42 to 0.37)	0.253	84.4%	0.002	0.435
Pyroglutamic acid	5	174/149	−0.64 (−1.76 to 0.49)	0.267	95.1%	<0.001	0.680
Serotonin	5	144/184	−0.26 (−0.76 to 0.23)	0.301	77.9%	0.001	0.575
Dimethylglycine	3	76/83	1.18 (−1.08 to 3.44)	0.305	97.0%	<0.001	0.543
l-Carnitine	3	138/109	−0.40 (−1.18 to 0.37)	0.308	88.2%	<0.001	0.587
Isocitric acid	4	122/108	1.06 (−0.98 to 3.10)	0.308	97.4%	<0.001	0.186
Choline	4	134/125	0.75 (−0.72 to 2.22)	0.316	96.2%	<0.001	0.253
Glycoursodeoxycholic acid	3	144/130	0.19 (−0.19 to 0.58)	0.327	53.5%	0.116	0.800
Ethanolamine	5	208/170	−0.94 (−2.84 to 0.97)	0.336	98.1%	<0.001	0.613
*cis*-Aconitic acid	3	76/83	1.42 (−1.50 to 4.35)	0.341	97.9%	<0.001	0.304
1-Methylhistidine	4	161/111	−0.33 (−1.01 to 0.35)	0.342	85.7%	<0.001	0.540
l-Lactic acid	5	180/150	−0.37 (−1.23 to 0.49)	0.400	92.3%	<0.001	0.731
Deoxycholic acid	3	144/130	−0.17 (−0.60 to 0.25)	0.427	61.1%	0.076	0.985
O-Phosphoethanolamine	4	161/111	−0.21 (−0.75 to 0.33)	0.448	78.3%	0.003	0.814
5-Hydroxylysine	3	135/86	0.73 (−1.22 to 2.68)	0.464	97.3%	<0.001	0.107
Citrulline	8	349/473	0.18 (−0.31 to 0.68)	0.466	89.3%	<0.001	0.533
l-Proline	6	216/155	−0.35 (−1.40 to 0.70)	0.510	94.9%	<0.001	0.068
l-alpha-aminobutyric acid	3	145/112	0.17 (−0.40 to 0.73)	0.563	77.4%	0.012	0.416
Sarcosine	4	144/105	−0.20 (−0.95 to 0.55)	0.602	86.5%	<0.001	0.794
Quinolinic acid	10	453/415	−0.06 (−0.32 to 0.20)	0.660	69.7%	<0.001	0.500
3-Hydroxybutyric acid	4	134/125	0.11 (−0.41 to 0.63)	0.670	74.6%	0.008	0.821
Betaine	4	128/133	0.26 (−0.99 to 1.51)	0.679	95.3%	<0.001	0.410
4-Hydroxyproline	5	170/130	−0.16 (−0.97 to 0.65)	0.701	90.3%	<0.001	0.794
l-Histidine	7	279/237	0.09 (−0.51 to 0.70)	0.763	90.2%	<0.001	0.479
l-Aspartic acid	7	262/211	0.09 (−0.53 to 0.72)	0.771	90.0%	<0.001	0.991
Phosphatidylcholine (32:0)	3	150/130	0.08 (−0.51 to 0.66)	0.799	80.6%	0.006	0.694
Hydroxykynurenine	6	261/300	0.03 (−0.19 to 0.25)	0.804	37.7%	0.155	0.615
l-Cystine	3	135/86	0.25 (−2.70 to 3.20)	0.870	98.5%	<0.001	0.435
l-Glutamic acid	7	271/245	0.06 (−0.73 to 0.86)	0.873	94.1%	<0.001	0.492
3-Aminoisobutanoic acid	4	161/111	−0.06 (−1.03 to 0.92)	0.911	93.0%	<0.001	0.932
beta-Alanine	5	170/130	−0.02 (−0.47 to 0.43)	0.944	70.5%	0.009	0.697
l-Arginine	9	404/498	−0.01 (−0.73 to 0.70)	0.970	95.2%	<0.001	0.578
Cholesterol	3	113/86	0.00 (−1.17 to 1.16)	0.997	92.3%	<0.001	0.867

*CI* confidence interval, *CON* control, *MDD* major depressive disorder, *N* number, *SMD* standardized mean difference

^a^*p* value for between-group effect sizes

^b^*p* value for heterogeneity calculated using a chi-square analysis

^c^*p* value for Egger’s test. A *p* < 0.1 is indicative of this asymmetry

Subgroup analysis based on antidepressant exposure revealed that antidepressant-free MDD (AF-MDD) patients had higher levels of l-proline and 3-hydroxybutyric acid and lower levels of creatinine, l-tryptophan, kynurenic acid, l-leucine, l-kynurenine, taurine, and l-histidine compared with controls. Antidepressant-treated MDD (AT-MDD) patients had decreased levels of glycine, hypoxanthine, kynurenic acid, l-asparagine, l-glutamine, l-serine, and l-tryptophan compared with controls (all *p* < 0.05; Supplementary Table 2). Interestingly, six of the seven differential metabolites identified in AT-MDD patients were shared among all MDD patients, whereas five of the nine differential metabolites identified in AF-MDD patients were unique to AF-MDD (Supplementary Fig. 4a). Furthermore, l-tryptophan and kynurenic acid were consistently downregulated, regardless of antidepressant exposure. There were no significant differences in the SMDs between biological samples (plasma versus serum; Supplementary Fig. 4b, Supplementary Table 3).

Sensitivity analysis stratified by mean age found 16 differential metabolites between adult MDD patients and controls aged > 18 years (Supplementary Table 4), two of which, phosphatidylcholine (32:1) and myo-inositol, were only significantly dysregulated in adult patients (Supplementary Fig. 4c). Sensitivity analysis stratified by analytic technique identified 20 differential metabolites in studies that used MS platforms, all of which were also identified using all analytic techniques (Supplementary Fig. 4d, Supplementary Table 5). In the meta-regression analysis, no significant associations between factors (sample size, proportion of females, mean age, and disease severity) and effect sizes (all meta-regression *p* > 0.05; Supplementary Table 6) were identified, except for a significant negative correlation between disease severity and effect size for l-tryptophan [slope = −0.040, 95% CI (−0.076, −0.005), *p* = 0.029; Supplementary Fig. 5], suggesting that patients with higher depression rating scores may have lower levels of l-tryptophan.

### Bioinformatics analysis of blood metabolites in MDD

**Fig. 1:**
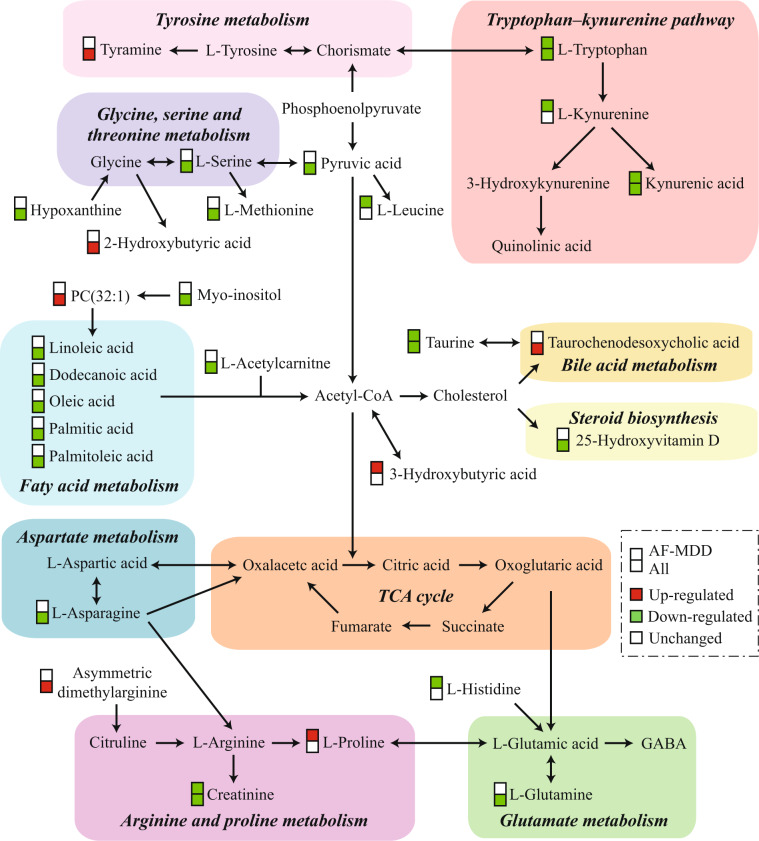
A simplified schematic diagram of the altered metabolic pathways in the blood of patients with MDD. For differential metabolites, boxes in red represent increased levels, boxes in green represent decreased levels, and boxes in white represent no significant change when compared with controls. Upper and lower boxes represent antidepressant-free major depressive disorder (AF-MDD) and all patients, respectively. Acetyl-CoA acetyl coenzyme A; GABA gamma-aminobutyric acid; MDD major depressive disorder; PC phosphatidylcholine; TCA tricarboxylic acid cycle.

**Fig. 2:**
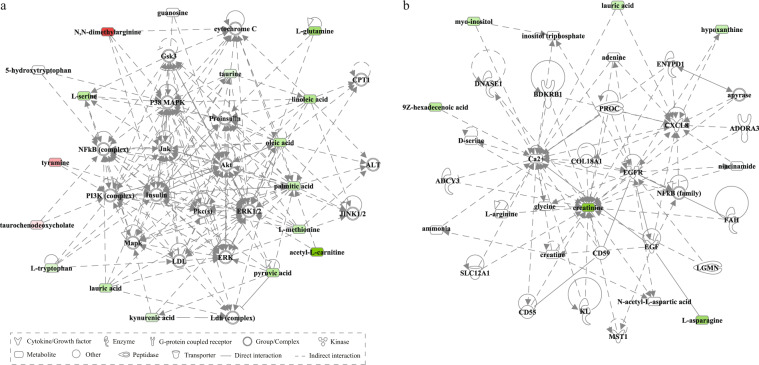
Altered networks associated with the differential metabolites in the blood of patients with MDD. **a** This network is associated with “developmental disorder, hereditary disorder, and metabolic disease” (score 42, 15 differential metabolites). **b** This network is associated with “small molecule biochemistry, increased levels of creatinine, and cell signaling” (score 13, six differential metabolites). Differential metabolites highlighted in red represent increased levels, whereas metabolites highlighted in green represent decreased levels when compared with controls.

Bioinformatics analysis was performed separately for three groups of differential metabolites (all MDD, AF-MDD, and AT-MDD patients). For all patients, metabolic pathway analysis using MetaboAnalyst identified 10 altered metabolic pathways; the top-ranked metabolic pathways were “nitrogen metabolism,” “aminoacyl-tRNA biosynthesis,” and “fatty acid biosynthesis.” Canonical pathway analysis in IPA identified 20 significantly disturbed canonical pathways; the top-ranked pathways were “tRNA charging,” “glycine betaine degradation,” and “l-serine degradation” (Table 2). To better understand the interactions between pathways, we summarized these pathways in a brief plot (Fig. 1). Network analysis in IPA revealed a significantly altered network for “developmental disorder, hereditary disorder, metabolic disease” (score 42), which was associated with 15 differential metabolites (Fig. 2a), and for “small molecule biochemistry, increased levels of creatinine, cell signaling” (score 13), which was associated with six differential metabolites (Fig. 2b).

**Table 2:** Altered pathways in the blood of patients with major depressive disorder.

Pathways	*p* value^a^
Metabolic pathways identified by MetaboAnalyst	
Nitrogen metabolism	<0.001
Aminoacyl-tRNA biosynthesis	<0.001
Fatty acid biosynthesis	0.001
Alanine, aspartate and glutamate metabolism	0.001
Linoleic acid metabolism	0.008
Glycine, serine and threonine metabolism	0.009
Cyanoamino acid metabolism	0.009
Cysteine and methionine metabolism	0.013
Taurine and hypotaurine metabolism	0.014
Arginine and proline metabolism	0.031
Canonical pathways identified by IPA	
tRNA charging	<0.001
Glycine betaine degradation	<0.001
l-Serine degradation	<0.001
Asparagine biosynthesis I	0.003
Palmitate biosynthesis I (animals)	0.006
Superpathway of methionine degradation	0.007
Cysteine biosynthesis III (mammalia)	0.010
NAD biosynthesis II (from tryptophan)	0.021
Folate transformations I	0.021
HIF1α signaling	0.024
Asparagine degradation I	0.035
Glutamine degradation I	0.035
Pyruvate fermentation to lactate	0.047
Glycine biosynthesis III	0.047
Alanine degradation III	0.047
Alanine biosynthesis II	0.047
Myo-inositol biosynthesis	0.047
l-Cysteine degradation II	0.047
Phosphatidylethanolamine biosynthesis III	0.047
Glycine biosynthesis I	0.047

^a^*p* values were calculated from hypergeometric tests in MetaboAnalyst, and from Fisher’s exact tests in Ingenuity pathways analysis

We then investigated the altered pathways and networks associated with antidepressant exposure. Respectively, five and nine metabolic pathways were significantly altered in AF-MDD and AT-MDD patients (Supplementary Table 7). “Aminoacyl-tRNA biosynthesis,” “nitrogen metabolism,” and “tryptophan metabolism” were shared pathways among both groups of patients. The canonical pathway analysis identified 4 and 45 significantly disturbed pathways for AF-MDD and AT-MDD patients, respectively (Supplementary Table 8), “tRNA charging” was a shared pathway. Network analysis revealed that “organismal injury and abnormalities, increased levels of creatinine, small molecule biochemistry” (score 27, nine differential metabolites) was a significantly altered network for AF-MDD (Supplementary Fig. 6a), and that “amino acid metabolism, cell-to-cell signaling and interaction, molecular transport” (score 21, seven differential metabolites) was significantly altered for AT-MDD (Supplementary Fig. 6b).

## Discussion

In the present study, we integrated the peripheral blood metabolic profiles from a large sample of MDD patients and controls and found that the levels of five metabolites were significantly elevated in the peripheral blood of MDD patients compared with controls, whereas the levels of 18 metabolites were reduced in MDD patients compared with controls. l-tryptophan and kynurenic acid were consistently downregulated in MDD patients, regardless of antidepressant exposure. Moreover, we explored the biological themes associated with these metabolic changes, based on pathway and network analyses, which suggested that several pathways involved in amino acid metabolism and lipid metabolism, especially the tryptophan–kynurenine pathway and fatty acid metabolism, were significantly perturbed in the peripheral blood of MDD patients.

Increasing investigations have sought to identify potential blood-based biomarkers of psychiatric diseases [34, 35], and our findings may facilitate further biomarker development for MDD. Among the altered metabolites, only tryptophan, kynurenic acid, and 25-hydroxyvitamin D were identified in studies that examined relatively large samples (>1000 participants), which may provide more credible estimates than smaller samples. Furthermore, we observed heterogeneity for the metabolic changes among the included studies, which could partially be explained by antidepressant exposure. Thus, antidepressant exposure should be considered in future metabolic biomarker studies. Subgroup analyses of antidepressant exposure found that only tryptophan and kynurenic acid were consistently downregulated in MDD patients, regardless of antidepressant exposure, and meta-regression analysis revealed that patients with higher depression scores might have lower levels of tryptophan, which is consistent with previous meta-analyses [21, 23]. These findings suggest that certain metabolic markers may be used to distinguish the MDD disease state and to monitor the therapeutic response [36, 37], with the most convincing evidence existing for l-tryptophan, followed by kynurenic acid.

Despite these findings, whether any of these metabolites can be used as biomarkers for MDD remains unresolved. Biomarkers in peripheral blood might not, in theory, reflect metabolic changes in the brain, which requires further direct evidence. Moreover, the utility of any individual metabolite biomarker for MDD practically remains unclear. For example, we reported that the area under the curve for tryptophan associated with MDD was 0.74, which remains inadequate for clinical practice, suggesting that diagnostic systems that include panels of differential metabolites are likely to result in better diagnostic efficacy than individual metabolites [38]. Therefore, more metabolic profiling studies are necessary to develop promising diagnostic systems. Given the heterogeneity of depression, such as concomitant somatic diseases, clinical variances should be addressed to improve the diagnostic performance. Because metabolomics studies face challenges from methodological sources of variance, the application of more rigorous experimental designs and processes will be necessary for future progress, as described in methodological reviews [39, 40].

Our integrated results also provided clues to the potential biological mechanisms that underly MDD. We found significantly disturbed amino acid metabolism in the peripheral blood of MDD patients. The levels of five amino acids (serine, methionine, asparagine, glutamine, and tryptophan) were decreased in the blood samples from all patients, and the levels of four amino acids (histidine, leucine, taurine, and tryptophan) were decreased in AF-MDD patients compared with controls. Pathway analyses revealed nitrogen metabolism and tRNA charging to be among the top-ranked pathways, reflecting perturbations in amino acid metabolism [41, 42]. Moreover, we found that the tryptophan–kynurenine pathway was the most enriched amino acid metabolism pathway. This pathway produces both neuroprotective (kynurenic acid) and neurotoxic (3-hydroxykynurenine and quinolinic acid) metabolites [43], and decreased levels of kynurenic acid have been reported to indicate increased neurotoxic burdens during in the course of depression, which could be reversed by physical exercise and electroconvulsive therapy [16, 44]. Furthermore, pro-inflammatory cytokines (e.g., interferon) have been demonstrated to mediate the enzymatic activity of the kynurenine pathway [45], resulting in decreased neuroprotective effects for kynurenic acid. This result is consistent with the findings that interferon-induced depression in ~50% of patients receiving interferon treatment [46] and that interferon treatment resulted in decreased levels of tryptophan and increased levels of kynurenine and quinolinic acid in the rodent brain [47, 48]. Finally, anti-inflammatory treatments have been reported to decrease depressive symptoms in patients [49]. Overall, these data suggest that the tryptophan–kynurenine pathway may be involved in depression by mediating inflammatory responses.

Other amino acid metabolism pathways, as summarized in Fig. 1, were also found to be enriched. One interpretation of these findings is that these pathways are involved in neurotransmission. Glutamine is a precursor of glutamate and gamma-aminobutyric acid [50], and altered levels of circulating glutamine may affect the gamma-aminobutyric acid levels in the brain [51]. Proline is synthesized from glutamic acid, and chronic hyperprolinemia can lead to reduced glutamate uptake, increased adenosine triphosphate catabolism, and increased pro-inflammatory cytokine levels [52–54]. Moreover, monoamine neurotransmitters may be involved in MDD, as tryptophan is the precursor for serotonin and tyrosine is the precursor for catecholamines. In addition to neurotransmission, these disturbed pathways have also been associated with energy metabolism. In addition to the downregulated amino acids, being glucogenic or ketogenic, other downregulated metabolites, including creatinine, hypoxanthine, and pyruvic acid, were associated with periphery energy dyshomeostasis [55, 56]. Thus, these results suggested that disturbed amino acid metabolism may contribute to depression by modulating neurotransmission and energy metabolism.

Our integrated results also suggested that lipid metabolism was dysregulated during MDD. Fatty acid biosynthesis was among the top-ranked metabolic pathways, and the levels of five fatty acids and l-acetylcarnitine were significantly decreased in the blood of MDD patients compared with controls. Fatty acid alterations may contribute to depression through several mechanisms, such as affecting cell membrane structure, biological stress, and inflammatory responses [57]. l-Acetylcarnitine plays a pivotal role in the transport of fatty acids into the mitochondria for beta-oxidation, and l-acetylcarnitine supplementation was reported to have antidepressive effects [58]. We also found increased levels of taurochenodesoxycholic acid, a bile acid formed from taurine in the liver, in the blood of MDD patients. Animal studies of depression have reported increased taurochenodesoxycholic acid levels and decreased taurine levels in the liver [59, 60], indicating that primary bile acid biosynthesis may be associated with MDD. Furthermore, we observed decreased levels of 25-hydroxyvitamin D in the present study, which was consistent with a previous meta-analysis [61]. 25-Hydroxyvitamin D is the primary form of vitamin D in the human body, and a deficiency in vitamin D has been associated with higher rates of suicide and the elevation of pro-inflammatory cytokines [62, 63]. Taken together, these findings suggested that alterations in lipid metabolism may play a key role in the pathogenesis of MDD.

Our network analysis also revealed that the mitogen-activated protein kinase (MAPK) signaling pathway, which includes *Mapk*, extracellular signal-regulated kinase 1/2, c-Jun N-terminal kinase (*Jnk*), *P38 MAPK*, and protein kinase C [64], and the phosphoinositide 3-kinase/protein kinase B (PI3K/AKT) signaling pathway, which includes *PI3K*, *Akt*, glycogen synthase kinase 3, and insulin [65], were both linked to the identified altered networks, indicating cross-talk between signaling pathways and differentially expressed metabolites. We also reported decreased peripheral levels of myo-inositol, which forms the structural basis for secondary messengers in the phosphoinositol system [66]. Overall, our data supported the potential involvement of signaling pathways in the peripheral metabolic changes observed in MDD patients.

This study has several limitations. First, without metabolic and clinical data from individual patients, the adjustment of potential confounders was not possible during our analysis. The integration of individual patient data is required in future studies. Second, the sample size was relatively small for certain metabolites in our analysis, resulting in low statistical power for those metabolites. Further studies with more patients are required to validate our findings. Third, we only included studies that reported MS-based or NMR-based techniques. This decision was made a priori because these techniques have higher accuracies and wider detection ranges than other techniques, which has revolutionized metabolite measurement [67]. Although this strategy is also used in other meta-analyses [26, 68], however, this limitation may also lead to selection bias. For example, previous studies utilizing high-performance liquid chromatography with fluorescence detection reported decreased or unchanged tryptophan levels in MDD patients compared with controls [69, 70]. Fourth, confidence in metabolite annotation and quantification remains a primary challenge for metabolomics studies [71, 72]; therefore, plausible metabolite candidates from the included studies may limit the generalization of our findings. Finally, during the process of metabolite identification and standardization across studies, a small number of metabolites were lost due to the use of non-standardized metabolite nomenclature.

### Summary

We identified differential metabolites in the peripheral blood of MDD patients, using a meta-analysis of curated metabolic characterization data from a large sample of patients. Subgroup analyses showed that antidepressant exposure was the most important source of heterogeneity. Pathway and network analyses revealed disturbances of amino acid and lipid metabolism, especially the tryptophan–kynurenine pathway and fatty acid metabolism, in the peripheral systems of MDD patients. This integrated approach may facilitate the development of biomarkers for MDD and help to determine the underlying molecular mechanisms associated with MDD. Future replication studies using larger sample sizes are necessary to confirm our findings.

## Supplementary information

Supplementary Materials

Supplementary Table 1

Supplementary Table 2

Supplementary Table 3

Supplementary Table 4

Supplementary Table 5

Supplementary Table 6

Supplementary Table 7

Supplementary Table 8

Supplementary Figure 1

Supplementary Figure 2

Supplementary Figure 3

Supplementary Figure 4

Supplementary Figure 5

Supplementary Figure 6

Supplementary Data
