# Supplementary material for: An integrated meta-analysis of peripheral blood metabolites and biological functions in major depressive disorder
Source: Mol Psychiatry. 2020 Jan 20;26(8):4265–76. doi: 10.1038/s41380-020-0645-4 (PMC8550972; doi:10.1038/s41380-020-0645-4)
Supplement: Supplementary file 1 — Supplementary Materials [file 41380_2020_645_MOESM1_ESM.docx]

**Supplementary Materials for:**

***An*** ***integrated meta-analysis of peripheral blood metabolites and*** ***biological functions in major depressive disorder***

Juncai Pu, Yiyun Liu, Hanping Zhang, Lu Tian, Siwen Gui, Yue Yu, Xiang Chen, Yue Chen, Lining Yang, Yanqin Ran, Xiaogang Zhong, Shaohua Xu, Xuemian Song, Lanxiang Liu, Peng Zheng, Haiyang Wang, Peng Xie

This article contains Tables 1–2, Figures 1–2, Supplementary Tables 1–8, Supplementary Figures 1–6, Supplementary Materials, and Supplementary Data.

**Supplementary Table 1** Summary of study characteristics for the included studies

**Supplementary Table 2** Subgroup analyses, according to antidepressant exposure

**Supplementary Table 3** Subgroup analyses, according to biological sample

**Supplementary Table 4** Sensitivity analyses, according to mean age

**Supplementary Table 5** Sensitivity analyses, according to analytic technique

**Supplementary Table 6** Meta-regression analyses of metabolites in the blood, comparing MDD patients and controls

**Supplemental Table 7** Altered metabolic pathways in the blood of patients with MDD, according to antidepressant exposure

**Supplemental Table 8** Significantly altered canonical pathways in the blood of patients with MDD, according to antidepressant exposure

**Supplementary Figure 1. Flowchart describing the identification of relevant studies.** After screening 13,542 citations from literature databases, 208 citations from metabolomics databases, and 777 citations from hand searches, a total of 1,762 full-text articles were reviewed. After screening these full-text articles, 527 studies were included in our online database MENDA. From these studies, we included 46 studies for further analysis.

**Supplementary Figure 2. The altered metabolites identified in the meta-analyses.** The x-axis shows the standardized mean difference (SMD). Hollow circles in the y-axis represent the altered metabolites, with darker fill colors representing smaller *p*-values for the overall effect and bigger circles representing larger sample sizes. Positive and negative SMD values indicate higher and lower levels of metabolites in the MDD group relative to the control group, respectively.

**Supplementary Figure 3**. **Forest plots showing the standard mean differences for meta-analyses of studies evaluating blood levels of metabolites**. The identified metabolites included **(a-w)** asymmetric dimethylarginine, tyramine, 2-hydroxybutyric acid, phosphatidylcholine (32:1), taurochenodesoxycholic acid, L-acetylcarnitine, creatinine, L-asparagine, L-glutamine, linoleic acid, pyruvic acid, palmitoleic acid, L-serine, oleic acid, myo-inositol, dodecanoic acid, L-methionine, hypoxanthine, palmitic acid, L-tryptophan, kynurenic acid, taurine, and 25-hydroxyvitamin D. The summary effect size is represented by the diamond. *CI*, confidence interval; *MDD*, major depressive disorder; *SMD*, standard mean differences.

**Supplementary Figure 4.** **Numbers of shared differential metabolites among the subgroup and sensitivity analyses. a** Venn diagram displaying the shared metabolites among antidepressant-free major depressive disorder (AF-MDD), antidepressant-treated major depressive disorder (AT-MDD), and all patients (All). **b** Venn diagram displaying shared metabolites among plasma, serum, and all biological samples. **c** Venn diagram displaying shared metabolites among patients aged > 18 years and all patients. **d** Venn diagram displaying shared metabolites among mass spectrometry (MS) platforms and all platforms.

**Supplementary Figure 5**. **Correlation between disease severity (17-item Hamilton Depression Rating Scale scores) and effect size for tryptophan**. Nodes represent the effect sizes for each study, the x-axis shows the 17-item Hamilton Depression Rating Scale (HAMD-17) scores, and the y-axis shows the summary effect sizes, i.e., the standard mean differences (SMDs).

**Supplementary Figure 6**. **Altered networks associated with the differential metabolites identified in the blood of antidepressant-free and antidepressant-treated patients with MDD. a** Altered networks identified in antidepressant-free patients, which are associated with organismal injury and abnormalities, increased levels of creatinine, and small molecule biochemistry (score 27, 9 differential metabolites). **b** Altered networks identified in antidepressant-treated patients, which are associated with amino acid metabolism, cell-to-cell signaling and interaction, and molecular transport (score 21, 7 differential metabolites). Differential metabolites highlighted in red represent increased levels, whereas metabolites highlighted in green represent decreased levels when compared with controls.

**Supplementary references for included articles (n = 46)**

1. Ali-Sisto T, Tolmunen T, Toffol E, et al. Purine metabolism is dysregulated in patients with major depressive disorder. Psychoneuroendocrinology 2016;70:25–32.

2. Ali-Sisto T, Tolmunen T, Viinamäki H, et al. Global arginine bioavailability ratio is decreased in patients with major depressive disorder. J Affect Disord 2018;229:145–51.

3. Baranyi A, Amouzadeh-Ghadikolai O, von Lewinski D, et al. Revisiting the tryptophan-serotonin deficiency and the inflammatory hypotheses of major depression in a biopsychosocial approach. PeerJ 2017;5:e3968.

4. Black CN, Bot M, Scheffer PG, et al. Oxidative stress in major depressive and anxiety disorders, and the association with antidepressant use; results from a large adult cohort. Psychol Med 2017;47(5):936–48.

5. Cho HJ, Savitz J, Dantzer R, et al. Sleep disturbance and kynurenine metabolism in depression. J Psychosom Res 2017;99:1–7.

6. Chu C, Wei H, Zhu W, et al. Decreased prostaglandin D2 levels in major depressive disorder are associated with depression-like behaviors. Int J Neuropsychopharmacol 2017;20(9):731–9.

7. DeWitt SJ, Bradley KA, Lin N, et al. A pilot resting-state functional connectivity study of the kynurenine pathway in adolescents with depression and healthy controls. J Affect Disord 2018;227:752–8.

8. Ding X, Yang S, Li W, et al. The potential biomarker panels for identification of major depressive disorder (MDD) patients with and without early life stress (ELS) by metabonomic analysis. PLoS One 2014;9(5):e97479.

9. Doolin K, Allers KA, Pleiner S, et al. Altered tryptophan catabolite concentrations in major depressive disorder and associated changes in hippocampal subfield volumes. Psychoneuroendocrinology 2018;95:8–17.

10. Epperson CN, Gueorguieva R, Czarkowski KA, et al. Preliminary evidence of reduced occipital GABA concentrations in puerperal women: a 1H-MRS study. Psychopharmacology 2006;186(3):425–33.

11. Grudet C, Malm J, Westrin A, et al. Suicidal patients are deficient in vitamin D, associated with a pro-inflammatory status in the blood. Psychoneuroendocrinology 2014;50:210–9.

12. Hill M, Řípová D, Mohr P, et al. Circulating C19 steroids and progesterone metabolites in women with acute depression and anxiety disorders. Horm Mol Biol Clin Investig 2016;26(3):153–64.

13. Hill MN, Miller GE, Carrier EJ, et al. Circulating endocannabinoids and N-acyl ethanolamines are differentially regulated in major depression and following exposure to social stress. Psychoneuroendocrinology 2009;34(8):1257–62.

14. Kageyama Y, Kasahara T, Morishita H, et al. Search for plasma biomarkers in drug-free patients with bipolar disorder and schizophrenia using metabolome analysis. Psychiatry Clin Neurosci 2017;71(2):115–23.

15. Kageyama Y, Kasahara T, Nakamura T, et al. Plasma nervonic acid is a potential biomarker for major depressive disorder: a pilot study. Int J Neuropsychopharmacol 2018;21(3):207–15.

16. Kawamura N, Shinoda K, Sato H, et al. Plasma metabolome analysis of patients with major depressive disorder. Psychiatry Clin Neurosci 2018;72(5):349–61.

17. Klumpers UM, Veltman DJ, Drent ML, et al. Reduced parahippocampal and lateral temporal GABAA-[11C]flumazenil binding in major depression: preliminary results. Eur J Nucl Med Mol Imaging 2010;37(3):565–74.

18. Kuwano N, Kato TA, Setoyama D, et al. Tryptophan-kynurenine and lipid related metabolites as blood biomarkers for first-episode drug-naïve patients with major depressive disorder: an exploratory pilot case-control study. J Affect Disord 2018;231:74–82.

19. Liu H, Ding L, Zhang H, et al. The metabolic factor kynurenic acid of kynurenine pathway predicts major depressive disorder. Front Psychiatry 2018;9:552.

20. Liu X, Zheng P, Zhao X, et al. Discovery and validation of plasma biomarkers for major depressive disorder classification based on liquid chromatography-mass spectrometry. J Proteome Res 2015;14(5):2322–30.

21. Meier TB, Drevets WC, Wurfel BE, et al. Relationship between neurotoxic kynurenine metabolites and reductions in right medial prefrontal cortical thickness in major depressive disorder. Brain Behav Immun 2016;53:39–48.

22. Milaneschi Y, Hoogendijk W, Lips P, et al. The association between low vitamin D and depressive disorders. Mol Psychiatry 2014;19(4):444–51.

23. Moaddel R, Shardell M, Khadeer M, et al. Plasma metabolomic profiling of a ketamine and placebo crossover trial of major depressive disorder and healthy control subjects. Psychopharmacology 2018;235(10):3017–30.

24. Nasca C, Bigio B, Lee FS, et al. Acetyl-l-carnitine deficiency in patients with major depressive disorder. Proc Natl Acad Sci U S A 2018;115(34):8627–32.

25. Ogawa S, Fujii T, Koga N, et al. Plasma L-tryptophan concentration in major depressive disorder: new data and meta-analysis. J Clin Psychiatry 2014;75(9):e906–15.

26. Ogawa S, Koga N, Hattori K, et al. Plasma amino acid profile in major depressive disorder: analyses in two independent case-control sample sets. J Psychiatr Res 2018;96:23–32.

27. Paige LA, Mitchell MW, Krishnan KR, et al. A preliminary metabolomic analysis of older adults with and without depression. Int J Geriatr Psychiatry 2007;22(5):418–23.

28. Pan JX, Xia JJ, Deng FL, et al. Diagnosis of major depressive disorder based on changes in multiple plasma neurotransmitters: a targeted metabolomics study. Transl Psychiatry 2018;8(1):130.

29. Paul-Savoie E, Potvin S, Daigle K, et al. A deficit in peripheral serotonin levels in major depressive disorder but not in chronic widespread pain. Clin J Pain 2011;27(6):529–34.

30. Petrov B, Aldoori A, James C, et al. Bipolar disorder in youth is associated with increased levels of vitamin D-binding protein. Transl Psychiatry 2018;8(1):61.

31. Quak J, Doornbos B, Roest AM, et al. Does tryptophan degradation along the kynurenine pathway mediate the association between pro-inflammatory immune activity and depressive symptoms? Psychoneuroendocrinology 2014;45:202–10.

32. Savitz J, Drevets WC, Smith CM, et al. Putative neuroprotective and neurotoxic kynurenine pathway metabolites are associated with hippocampal and amygdalar volumes in subjects with major depressive disorder. Neuropsychopharmacology 2015;40(2):463–71.

33. Selley ML. Increased (E)-4-hydroxy-2-nonenal and asymmetric dimethylarginine concentrations and decreased nitric oxide concentrations in the plasma of patients with major depression. J Affect Disord 2004;80(2–3):249–56.

34. Teraishi T, Hori H, Sasayama D, et al. (13)C-tryptophan breath test detects increased catabolic turnover of tryptophan along the kynurenine pathway in patients with major depressive disorder. Sci Rep 2015;5:15994.

35. Thesing CS, Bot M, Milaneschi Y, et al. Omega-3 and omega-6 fatty acid levels in depressive and anxiety disorders. Psychoneuroendocrinology 2018;87:53–62.

36. Umehara H, Numata S, Watanabe SY, et al. Altered KYN/TRP, Gln/Glu, and Met/methionine sulfoxide ratios in the blood plasma of medication-free patients with major depressive disorder. Sci Rep 2017;7(1):4855.

37. Veen C, Myint AM, Burgerhout KM, et al. Tryptophan pathway alterations in the postpartum period and in acute postpartum psychosis and depression. J Affect Disord 2016;189:298–305.

38. Woo HI, Chun MR, Yang JS, et al. Plasma amino acid profiling in major depressive disorder treated with selective serotonin reuptake inhibitors. CNS Neurosci Ther 2015;21(5):417–24.

39. Wu Y, Zhong X, Mai N, et al. Kynurenine pathway changes in late-life depression. J Affect Disord 2018;235:76–81.

40. Wurfel BE, Drevets WC, Bliss SA, et al. Serum kynurenic acid is reduced in affective psychosis. Transl Psychiatry 2017;7(5):e1115.

41. Xu HB, Fang L, Hu ZC, et al. Potential clinical utility of plasma amino acid profiling in the detection of major depressive disorder. Psychiatry Res 2012;200(2–3):1054–7.

42. Young KD, Drevets WC, Dantzer R, et al. Kynurenine pathway metabolites are associated with hippocampal activity during autobiographical memory recall in patients with depression. Brain Behav Immun 2016;56:335–42.

43. Zheng P, Gao HC, Li Q, et al. Plasma metabonomics as a novel diagnostic approach for major depressive disorder. J Proteome Res 2012;11(3):1741–8.

44. Zhou X, Liu L, Lan X, et al. Polyunsaturated fatty acids metabolism, purine metabolism and inosine as potential independent diagnostic biomarkers for major depressive disorder in children and adolescents. Mol Psychiatry 2019;24(10):1478–88.

45. Zhou Y, Zheng W, Liu W, et al. Antidepressant effect of repeated ketamine administration on kynurenine pathway metabolites in patients with unipolar and bipolar depression. Brain Behav Immun 2018;74:205–12.

46. Zhou Y, Zheng W, Liu W, et al. Cross-sectional relationship between kynurenine pathway metabolites and cognitive function in major depressive disorder. Psychoneuroendocrinology 2019;101:72–9.

**Supplementary Methods**

**Identification of relevant studies**

To identify relevant literature, the following steps were used. First, we included potential studies identified in our existing metabolite-disease association database, called the metabolite network of depression database (MENDA; http://menda.cqmu.edu.cn:8080/index.php), which has been described in detail elsewhere [1]. Briefly, we collected all available studies that investigated metabolic changes that were identified by the PubMed (http://www.ncbi.nlm.nih.gov/pubmed), Cochrane Library (http://onlinelibrary.wiley.com/cochranelibrary/search), Embase (http://www.embase.com), Web of Science (http://www.webofknowledge.com), PsycINFO (https://www.ebscohost.com/nursing/products/psycinfo), Human Metabolome Database (http://www.hmdb.ca), MetaboLights (http://www.ebi.ac.uk/metabolights), Metabolomics Workbench (http://www.metabolomicsworkbench.org), MetabolomeXchange (http://www.metabolomexchange.org/site), and Omics Discovery Index (http://www.omicsdi.org/home) databases, as of 20 March 2018. Studies that performed metabolic characterizations during current depressed states or following antidepressant treatments were included. Additional eligible studies were screened from the citation lists of the identified studies. Studies of both human participants (patients with depressive disorders or depressive symptoms) and animal models were included. For human research, we excluded studies in which more than 20% of patients had a primary diagnosis of other psychiatric disorders, such as bipolar disorder, schizophrenia, anxiety disorder, and substance-related disorder. We also excluded studies in which more than 20% of patients were remitted or in which the average depression score of the patient group was relatively low, e.g., 17-item Hamilton Depression Rating Scale (HAMD-17) < 8, or Montgomery-Asberg Depression Rating Scale (MADRS) score < 7 [2, 3]. The analytical platforms were restricted to metabolomics technologies (e.g. nuclear magnetic resonance and mass spectrometer based platforms) [4, 5]. We also included studies that use magnetic resonance spectroscopy (MRS) for living brain research [6]. Studies that used technologies than those previously described, such as high-performance liquid chromatography, gas chromatography, and enzyme-linked immunosorbent assay, were excluded. We excluded studies that reported duplicated samples using the same statistical strategies, other types of reports (review, case report, protocol, commentary, editorial), and meeting abstracts. A total of 11,955 citations from these databases were identified, and 464 studies were included in MENDA.

Second, we updated the search results retrieved from PubMed, Cochrane Library, Embase, Web of Science, and PsycINFO in January 2019 and screened for eligible studies using the same strategy, resulting in 527 included studies from 1,762 full-text articles.

Finally, we excluded studies from among the 527 potentially eligible studies using the following criteria. Animal studies (n = 216) and MRS research (n = 182) were excluded. We only considered studies performed on serum and plasma samples, whereas studies profiling other tissues were excluded due to limited data (n = 32). Human studies that recruited patients diagnosed with MDD, based on standardized diagnostic criteria (Diagnostic and Statistical Manual of Mental Disorders, 3rd, 4th, or 5th edition; and the International Classification of Diseases, 9th or 10th edition) or consensus expert evaluations confirming the diagnosis of depression, were included [7]. Studies that recruited patients with depressive symptoms were excluded (n = 18). To avoid the influence of other diseases on metabolic characterization, studies in which all patients had concurrent diagnoses of other psychiatric or physical diseases were excluded (n = 10). In addition, we also excluded studies that lacked control of interest (n = 10), secondary analyses (n = 6), were unable to extract any data (n = 5), reported no diagnosis available (n = 1), or were published in a language other than English (n = 1). The full lists of all excluded studies (1,235 for the first and second steps, and 481 for the last step) are provided in Supplementary Data.

**Data curation**

Candidate metabolites were selected if their concentrations were reported in the original reports. We excluded the ratio of two metabolites, e.g. kynurenine/tryptophan ratio. When metabolite synonyms were encountered, we compared each metabolite name against standard databases, including the Human Metabolome Database (http://www.hmdb.ca/), Kyoto Encyclopedia of Genes and Genomes (KEGG, https://www.genome.jp/kegg/), and PubChem (https://pubchem.ncbi.nlm.nih.gov/). For intervention or follow-up studies that reported both baseline and post-treatment (or follow-up) data for MDD patients, we only considered baseline data.

**Raw data processing**

For metabolomics studies, researchers analyzed raw data (metabolite concentration tables) for hundreds or thousands of metabolites. However, most authors only reported the concentrations of the top-ranked metabolites in their articles. For studies in which metabolite concentration tables (concentration data of each metabolite for each patient) were available, we calculated the mean and standard deviation values for unreported metabolites. Metabolites present in less than 80% of samples were excluded [8].

**Disease severity assessment**

Because several different depression rating scales were used in the included studies, we converted average depression scores into HAMD-17 scale scores using the transformation table available at http://www.ids-qids.org/interpretation.html. This table provided a conversion algorithm between HAMD-17, HAMD-21, HAMD-24, Beck Depression Inventory (BDI), MADRS, and 30-item Inventory of Depressive Symptoms – Self Report (IDS-SR) scores, based on previous studies [9, 10]. Studies that used scales that we could not transform into HAMD-17 scores were excluded from this analysis [11].

**Bioinformatics analysis**

Pathway analysis is a method for testing whether molecules of interest are associated with certain phenotypes, with a *p*-value describing the probability that the association between the uploaded molecules and a predefined biological pathway can be explained by chance alone [12]. In this study, both metabolic pathway analysis, using MetaboAnalyst 4.0 [13, 14], and canonical pathway analysis, using Ingenuity Pathways Analysis (IPA, http://www.ingenuity.com), were performed to identify significantly altered pathways. For metabolic pathway analysis, the hypergeometric test is the default statistical approach for calculating *p*-values, and ‘Homo sapiens (KEGG)’ was used as the backend pathway library. For canonical pathway analysis, the Fisher’s exact test is used to calculate *p*-values, and Ingenuity Pathway Knowledge Base in IPA was used as the backend pathway library. For both analyses, default parameters were used, and statistical significance was set at a *p*-value < 0.05.

Network analysis is a method for elucidating the interactions between uploaded molecules and other molecules in omics research. Two broad methods have been proposed, including evidence-based molecular networks, which rely on prior knowledge, and statistically inferred networks, which identifies sets of co-expressed molecules [15, 16]. In this study, evidence-based network analysis was performed, using IPA, to identify potential molecular networks according to literature-based interactions among uploaded metabolites and other biological molecules (genes, proteins) within the Ingenuity Pathway Knowledge Base. This method has been used in our previous metabolomics studies [17–19]. Networks were ranked based on a score that is calculated using the right-tailed Fisher’s exact test, with a cutoff of 35 molecules per network. Higher scores and more uploaded metabolites represented higher relevance for the identified networks. Lines between molecules in the network indicate a biomolecular relationship.

**References for supplementary methods**

1. Pu J, Yu Y, Liu Y, et al. MENDA: a comprehensive curated resource of metabolic characterization in depression. Brief Bioinform. 2019. Epub ahead of print 03 June 2019; doi: 10.1093/bib/bbz055.

2. Hamilton M. A rating scale for depression. J Neurol Neurosurg Psychiatry. 1960;23:56–62.

3. Montgomery SA, Asberg M. A new depression scale designed to be sensitive to change. Br J Psychiatry. 1979;134:382–9.

4. Wishart DS. Emerging applications of metabolomics in drug discovery and precision medicine. Nat Rev Drug Discov. 2016;15(7):473–84.

5. Marshall DD, Powers R. Beyond the paradigm: Combining mass spectrometry and nuclear magnetic resonance for metabolomics. Prog Nucl Magn Reson Spectrosc. 2017;100:1–16.

6. Henning A. Proton and multinuclear magnetic resonance spectroscopy in the human brain at ultra-high field strength: A review. Neuroimage. 2018;168:181–98.

7. Moriguchi S, Takamiya A, Noda Y, et al. Glutamatergic neurometabolite levels in major depressive disorder: a systematic review and meta-analysis of proton magnetic resonance spectroscopy studies. Mol Psychiatry. 2019;24(7):952–64.

8. Bozek K, Wei Y, Yan Z, et al. Exceptional evolutionary divergence of human muscle and brain metabolomes parallels human cognitive and physical uniqueness. PLoS Biol. 2014;12(5):e1001871.

9. Rush AJ, Trivedi MH, Ibrahim HM, et al. The 16-Item Quick Inventory of Depressive Symptomatology (QIDS), clinician rating (QIDS-C), and self-report (QIDS-SR): a psychometric evaluation in patients with chronic major depression. Biol Psychiatry. 2003;54(5):573–83.

10. Trivedi MH, Rush AJ, Ibrahim HM, et al. The Inventory of Depressive Symptomatology, Clinician Rating (IDS-C) and Self-Report (IDS-SR), and the Quick Inventory of Depressive Symptomatology, Clinician Rating (QIDS-C) and Self-Report (QIDS-SR) in public sector patients with mood disorders: a psychometric evaluation. Psychol Med. 2004;34(1):73–82.

11. Cipriani A, Furukawa TA, Salanti G, et al. Comparative efficacy and acceptability of 21 antidepressant drugs for the acute treatment of adults with major depressive disorder: a systematic review and network meta-analysis. Lancet. 2018;391:1357–66.

12. Draghici S, Khatri P, Tarca AL, et al. A systems biology approach for pathway level analysis. Genome Res. 2007;17(10):1537–45.

13. Xia J, Wishart DS. MetPA: a web-based metabolomics tool for pathway analysis and visualization. Bioinformatics. 2010;26(18):2342–4.

14. Chong J, Soufan O, Li C, et al. MetaboAnalyst 4.0: towards more transparent and integrative metabolomics analysis. Nucleic Acids Res. 2018;46(W1):W486–94.

15. Oulas A, Minadakis G, Zachariou M, Sokratous K, Bourdakou MM, Spyrou GM. Systems Bioinformatics: increasing precision of computational diagnostics and therapeutics through network-based approaches. Brief Bioinform. 2019;20(3):806–24.

16. Pu J, Liu X, Liu Y, et al. Sex-specific plasma metabolome signatures in major depressive disorder. Psychiatry Clin Neurosci. 2019;73(11):713–4.

17. Zhou X, Liu L, Lan X, et al. Polyunsaturated fatty acids metabolism, purine metabolism and inosine as potential independent diagnostic biomarkers for major depressive disorder in children and adolescents. Mol Psychiatry. 2019;24(10):1478–88.

18. Liu L, Zhou X, Zhang Y, et al. Hippocampal metabolic differences implicate distinctions between physical and psychological stress in four rat models of depression. Transl Psychiatry. 2018;8(1):4.

19. Yang L, Pu J, Liu L, et al. Integrated metabolomics and proteomics analysis revealed second messenger system disturbance in hippocampus of chronic social defeat stress rat. Front Neurosci. 2019;13:247.
