# Supplementary material for: An integrated meta-analysis of peripheral blood metabolites and biological functions in major depressive disorder
Source: Mol Psychiatry. 2020 Jan 20;26(8):4265–76. doi: 10.1038/s41380-020-0645-4 (PMC8550972; doi:10.1038/s41380-020-0645-4)
Supplement: Supplementary file 2 — Supplementary Table 1 [file 41380_2020_645_MOESM2_ESM.docx]

| **Supplementary Table 1** Summary of study characteristics for the included studies | | | | | | | | | | | | | | |
| --- | --- | --- | --- | --- | --- | --- | --- | --- | --- | --- | --- | --- | --- | --- |
| **Study** | **Biological sample** | **Recruiting area** | **Study design** | **Patients (n)** | **Controls (n)** | **Female (%)** | **Age** | **Drug free in patients (period)** | **Diagnostic criteria** | **Severity measure** | **Severity** | **Platform** | **Storage**  **temperature** | **Raw data^a^** |
| Ali-Sisto T 2016 (1) | Serum | Finland | Case-control | 99 | 253 | 52.6% | 50.8 | No | DSM-IV | BDI | 29.0 | MS | −70 °C | NA |
| Ali-Sisto T 2018 (2) | Serum | Finland | Case-control | 99 | 253 | 52.6% | 50.8 | No | DSM-IV | BDI | 29.0 | MS | −70 °C | NA |
| Baranyi A 2017 (3) | Serum | Austria | Case-control | 71 | 48 | 34.5% | 47.9 | No | Expert evaluation | BDI | 24.5 | MS | NR | NA |
| Black CN 2017 (4) | Plasma | Netherlands | Cohort | 316 | 562 | NA | NA | No | DSM-IV | IDS | NA | MS | −80 °C | NA |
| Cho HJ 2017 (5) | Serum | USA | Case-control | 68 | 66 | 72.4% | 34.3 | Yes (4 weeks) | DSM-IV-TR | MADRS | 27.4 | MS | −80 °C | NA |
| Chu CL 2017 (6) | Plasma | China | Case-control | 32 | 30 | 58.1% | 36.4 | Yes (4 weeks) | DSM-IV | HDRS-17 | 22.6 | MS | −80 °C | NA |
| DeWitt SJ 2018 (7) | Plasma | USA | Case-control | 14 | 7 | 71.4% | 16.7 | Yes (3 months) | DSM-IV-TR | CDRS-R | [≥](http://www.baidu.com/link?url=_p_6bJEdxR8PW9eIodY7L4sFphb3G4lBFgd_me0OOC2ozr6uKYNciIm6xYMQw-nQatV6cjdCfb0QLEpw7-nb4q)40 | MS | NR | NA |
| Ding XH 2014 (8) | Plasma | China | Case-control | 46 | 25 | NA | 28.8 | NA | DSM-IV | SDS | 53.0 | MS | −80 °C | NA |
| Doolin K 2018 (9) | Plasma | Ireland | Case-control | 74 | 37 | 59.5% | 32.2 | No | DSM-IV | HDRS-17 | 23.5 | MS | −80 °C | NA |
| Epperson CN 2006 (10) | Plasma | USA | Case-control | 9 | 14 | 100.0% | 30.6 | Yes (9 months) | DSM-III-R | HDRS-19 | 20.6 | MS | NR | NA |
| Grudet C 2014 (11) | Serum | Sweden | Case-control | 17 | 14 | 48.4% | 34.1 | Yes (4 weeks) | DSM-IV | MADRS | 25.0 | MS | −80 °C | NA |
| Hill M 2016 (12) | Plasma | Czech | Case-control | 22 | 17 | 100.0% | 18-45 | Yes | ICD-10 | CGI | [**≥**](http://www.baidu.com/link?url=_p_6bJEdxR8PW9eIodY7L4sFphb3G4lBFgd_me0OOC2ozr6uKYNciIm6xYMQw-nQatV6cjdCfb0QLEpw7-nb4q)4 | MS | −80 °C | NA |
| Hill MN 2009 (13) | Serum | USA | Case-control | 15 | 15 | 100.0% | 25.2 | Yes (6 months) | DSM-IV | HDRS-17 | 18.9 | MS | −70 °C | NA |
| Kageyama Y 2017a (14) | Plasma | Japan | Case-control | 9 | 19 | 53.6% | 37.1 | Yes (2 weeks) | DSM-IV | HDRS-21 | 11.2 | MS | −80 °C | Available |
| Kageyama Y 2017b cohort1 (15) | Plasma | Japan | Case-control | 9 | 19 | 53.6% | 37.1 | Yes (2 weeks) | DSM-IV | HDRS-21 | 11.2 | MS | −80 °C | Available |
| Kageyama Y 2017b cohort2 (15) | Plasma | Japan | Case-control | 45 | 90 | 51.9% | 49.6 | No | DSM-IV | HDRS-21 | 17.0 | MS | −80 °C | NA |
| Kawamura N 2018 (16) | Plasma | Japan | Case-control | 34 | 31 | 55.4% | 38.4 | No | DSM-IV-TR | CES-D | 31.6 | MS | NR | NA |
| Klumpers UMH 2010 (17) | Plasma | Netherlands | Case-control | 10 | 6 | 50.0%^b^ | 34.8^b^ | Yes (3 months) | DSM-IV | MADRS | 26.9^b^ | MS | −70 °C | NA |
| Kuwano N 2018 (18) | Plasma | Japan | Case-control | 15 | 19 | 44.1% | 30.2 | Yes | DSM-IV-TR | BDI | 33.1 | MS | −80 °C | NA |
| Liu HY 2018 (19) | Plasma | China | Case-control | 33 | 23 | 60.7% | 28.8 | Yes (1 week) | DSM-V | HDRS-24 | 31.7 | MS | −80 °C | NA |
| Liu XY 2015 cohort1 (20) | Plasma | China | Case-control | 60 | 59 | 49.6% | 43.2 | Yes | DSM-IV | HDRS-17 | 25.0 | MS | −80 °C | Available |
| Liu XY 2015 cohort2 (20) | Plasma | China | Case-control | 75 | 52 | 48.0% | 35.1 | No | DSM-IV | HDRS-17 | 24.2 | MS | −80 °C | Available |
| Meier TB 2016 (21) | Serum | USA | Case-control | 65 | 74 | 68.3%^c^ | 32.8^c^ | Yes (4 weeks) | DSM-IV-TR | MADRS | 27.6^c^ | MS | −80 °C | NA |
| Milaneschi Y 2014 (22) | Serum | Netherlands | Cohort | 1102 | 494 | 65.2% | 40.7 | No | DSM-IV | IDS | 32.4 | MS | −80 °C | NA |
| Moaddel R 2018 (23) | Plasma | USA | Case-control | 29 | 25 | 60.7%^d^ | 34.8^d^ | Yes (2 weeks) | DSM-IV | HDRS-17 | 21.6^d^ | MS | −80 °C | NA |
| Nasca C 2018 (24) | Plasma | USA | Case-control | 71 | 45 | 50.0% | 39.8 | No | DSM-IV | HDRS-17 | 20.2 | MS | NR | NA |
| Ogawa S 2014 (25) | Plasma | Japan | Case-control | 66 | 82 | 57.4% | 43.9 | No | DSM-IV | HDRS-17 | 14.3 | MS | −20 °C | NA |
| Ogawa S 2018 (26) | Plasma | Japan | Case-control | 51 | 65 | 59.5% | 43.8 | No | DSM-IV | HDRS-17 | 14.6 | MS | −80 °C | NA |
| Paige LA 2007 (27) | Plasma | USA | Case-control | 9 | 10 | 47.4% | 72.2 | No | DSM-III | MADRS | [**≥**](http://www.baidu.com/link?url=_p_6bJEdxR8PW9eIodY7L4sFphb3G4lBFgd_me0OOC2ozr6uKYNciIm6xYMQw-nQatV6cjdCfb0QLEpw7-nb4q)14 | MS | NR | NA |
| Pan JX 2018 cohort1 (28) | Plasma | China | Case-control | 50 | 50 | 51.0% | 37.6 | Yes | DSM-IV-TR | HDRS-17 | 24.6 | MS | −80 °C | NA |
| Pan JX 2018 cohort2 (28) | Plasma | China | Case-control | 49 | 40 | 49.4% | 37.3 | No | DSM-IV-TR | HDRS-17 | 23.3 | MS | −80 °C | NA |
| Paul-Savoie E 2011 (29) | Plasma | Canada | Case-control | 17 | 57 | 70.3% | 43.7 | No | DSM-IV | BDI | 29.7 | MS | NR | NA |
| Petrov B 2018 (30) | Serum | USA | Case-control | 11 | 13 | NA | 14.0 | NA | DSM-IV | NA | NA | MS | NR | NA |
| Quak J 2014 (31) | Plasma | Netherlands | Cohort | 1042 | 1770 | 66.6% | 41.9 | No | DSM-IV | IDS | 32.7 | MS | −80 °C | NA |
| Savitz J 2015 (32) | Serum | USA | Case-control | 29 | 20 | 69.4% | 35.8 | Yes (3 weeks) | DSM-IV-TR | HDRS-24 | 27.1 | MS | −80 °C | NA |
| Selley ML 2004 (33) | Plasma | Australia | Case-control | 25 | 25 | 50.0% | 46.6 | Yes | DSM-IV | NR | NR | MS | −70 °C | NA |
| Teraishi T 2015 (34) | Plasma | Japan | Case-control | 18 | 24 | 52.4% | 42.9 | No | DSM-IV | HDRS-21 | 12.6 | MS | NR | NA |
| Thesing CS 2018 (35) | Plasma | Netherlands | Cohort | 304 | 634 | 62.6% | 41.6 | No | DSM-IV | IDS | 31.8 | NMR | −85 °C | NA |
| Umehara H 2017 (36) | Plasma | Japan | Case-control | 33 | 33 | 69.7% | 46.8 | Yes | DSM-IV | HDRS-17 | 21.0 | MS | −80 °C | Available |
| Veen C 2016 (37) | Serum | Netherlands | Case-control | 23 | 29 | 100.0% | 32.4 | Yes | DSM-IV-TR | EPDS | 19.0 | MS | −80 °C | NA |
| Woo HI 2015 (38) | Plasma | Korea | Case-control | 68 | 22 | 77.8% | 65.7 | No | DSM-IV | HDRS-17 | 19.2 | MS | NR | NA |
| Wu YJ 2018 (39) | Serum | China | Case-control | 170 | 135 | 72.1% | 66.7 | No | DSM-IV | HDRS-17 | 11.5 | MS | −80 °C | NA |
| Wurfel BE 2017 (40) | Serum | USA | Case-control | 35 | 92 | 61.4% | 34.1 | No | DSM-IV | HDRS-25 | 35.0 | MS | −80 °C | NA |
| Xu HB 2012 (41) | Plasma | China | Case-control | 26 | 25 | 68.6% | 32.3 | Yes | DSM-IV | HDRS-17 | 24.2 | MS | −80 °C | NA |
| Young KD 2016 (42) | Serum | USA | Case-control | 35 | 25 | 61.7% | 36.4 | Yes (3 weeks) | DSM-IV-TR | HDRS-21 | 19.3 | MS | −80 °C | NA |
| Zheng P 2012 (43) | Plasma | China | Case-control | 58 | 42 | 60.0% | 33.2 | Yes | DSM-IV | HDRS-17 | 22.4 | NMR | −80 °C | Available |
| Zhou XY 2018 (44) | Plasma | China | Case-control | 84^e^ | 50^e^ | 48.5% | 15.7 | No^e^ | DSM-IV | HDRS-17 | 22.5 | MS | −80 °C | Available |
| Zhou YL 2018 (45) | Serum | China | Case-control | 84 | 60 | 48.6% | 33.6 | No | DSM-V | MADRS | 31.2 | MS | −80 °C | NA |
| Zhou YL 2019 (46) | Serum | China | Case-control | 146 | 72 | 46.3% | 33.4 | No | DSM-V | HDRS-17 | 22.46 | MS | −80 °C | NA |
| Note: Full references for all studies are given in Supplementary Materials.  ^a^ Raw metabolite concentration tables were used to generate mean and standard deviation values for unreported metabolites, if available. Metabolites present in less than 80% of samples were excluded.  ^b^ Data from 11 patients and 9 controls  ^c^ Data from 73 patients and 91 controls  ^d^ Data from 35 patients and 26 controls  ^e^ The patient group included 52 drug-free patients and 32 drug-treated patients. In the random effects models, the control group was divided into two groups (a control group for drug-free patients, n = 25; and a control group for drug-treated patients, n = 25)  *BDI* Beck Depression Inventory, *CES-D* Center for Epidemiologic Studies Depression Scale, *CGI* Clinical Global Impressions, *DSM* Diagnostic and Statistical Manual of Mental Disorders, *EPDS*, Edinburgh Postnatal Depression Scale, *HAMD* Hamilton Rating Scale for Depression, *ICD* International Classification of Diseases, *IDS* self-report Inventory of Depressive Symptomatology, *MADRS* Montgomery-Asberg Depression Rating Scale, *MS* mass spectrometry, *NA* not applicable, *NMR* nuclear magnetic resonance, *NR* not reported | | | | | | | | | | | | | | |
