# Supplementary material for: An integrated meta-analysis of peripheral blood metabolites and biological functions in major depressive disorder
Source: Mol Psychiatry. 2020 Jan 20;26(8):4265–76. doi: 10.1038/s41380-020-0645-4 (PMC8550972; doi:10.1038/s41380-020-0645-4)
Supplement: Supplementary file 3 — Supplementary Table 2 [file 41380_2020_645_MOESM3_ESM.docx]

| **Supplementary Table 2** Subgroup analyses, according to antidepressant exposure | | | | | | |
| --- | --- | --- | --- | --- | --- | --- |
| **Metabolites** | **Antidepressant free** | **No. of comparisons** | **SMD (95% CI)** | ***p-*Value^a^**  **(overall)** | ***I*^2^** | ***p-*Value^b^**  **(heterogeneity)** |
| 1-Methylhistidine | Yes | … | … | … | … | … |
|  | No | … | … | … | … | … |
| 25-Hydroxyvitamin D | Yes | … | … | … | … | … |
|  | No | … | … | … | … | … |
| 2-Hydroxybutyric acid | Yes | … | … | … | … | … |
|  | No | … | … | … | … | … |
| 3-Aminoisobutanoic acid | Yes | … | … | … | … | … |
|  | No | … | … | … | … | … |
| 3-Hydroxybutyric acid | Yes | 3 | 0.32 (0.03 to 0.61) | 0.032 | 1.9% | 0.361 |
|  | No | … | … | … | … | … |
| 4-Hydroxyproline | Yes | 3 | 0.24 (−0.10 to 0.57) | 0.163 | 0.0% | 0.837 |
|  | No | … | … | … | … | … |
| 5-Hydroxylysine | Yes | … | … | … | … | … |
|  | No | … | … | … | … | … |
| Adenosine diphosphate | Yes | … | … | … | … | … |
|  | No | … | … | … | … | … |
| Aminoadipic acid | Yes | 3 | −0.17 (−0.57 to 0.23) | 0.408 | 43.2% | 0.172 |
|  | No | … | … | … | … | … |
| Arachidonic acid | Yes | … | … | … | … | … |
|  | No | … | … | … | … | … |
| Asymmetric dimethylarginine | Yes | … | … | … | … | … |
|  | No | … | … | … | … | … |
| beta-Alanine | Yes | 3 | 0.03 (−0.36 to 0.42) | 0.863 | 24.7% | 0.265 |
|  | No | … | … | … | … | … |
| Betaine | Yes | 3 | −0.24 (−1.23 to 0.76) | 0.642 | 90.2% | <0.001 |
|  | No | … | … | … | … | … |
| Capric acid | Yes | … | … | … | … | … |
|  | No | … | … | … | … | … |
| Cholesterol | Yes | … | … | … | … | … |
|  | No | … | … | … | … | … |
| Choline | Yes | 3 | −0.20 (−0.57 to 0.16) | 0.277 | 32.5% | 0.227 |
|  | No | … | … | … | … | … |
| cis-Aconitic acid | Yes | … | … | … | … | … |
|  | No | … | … | … | … | … |
| Citric acid | Yes | 3 | −0.25 (−0.84 to 0.34) | 0.405 | 72.1% | 0.028 |
|  | No | … | … | … | … | … |
| Citrulline | Yes | 4 | −0.24 (−0.76 to 0.28) | 0.364 | 68.7% | 0.022 |
|  | No | 4 | 0.60 (−0.21 to 1.40) | 0.146 | 94.0% | <0.001 |
| Creatine | Yes | 4 | −0.19 (−0.51 to 0.13) | 0.242 | 43.0% | 0.154 |
|  | No | … | … | … | … | … |
| Creatinine | Yes | 4 | −0.78 (−1.30 to −0.25) | 0.004 | 76.7% | 0.005 |
|  | No | … | … | … | … | … |
| Deoxycholic acid | Yes | … | … | … | … | … |
|  | No | … | … | … | … | … |
| Dimethylglycine | Yes | … | … | … | … | … |
|  | No | … | … | … | … | … |
| Dodecanoic acid | Yes | … | … | … | … | … |
|  | No | … | … | … | … | … |
| Ethanolamine | Yes | … | … | … | … | … |
|  | No | 3 | −1.22 (−5.37 to 2.93) | 0.565 | 99.0% | <0.001 |
| Gamma-Aminobutyric acid | Yes | 3 | 0.65 (−1.24 to 2.54) | 0.501 | 96.1% | <0.001 |
|  | No | 4 | 0.93 (−0.41 to 2.27) | 0.173 | 94.7% | <0.001 |
| Gluconic acid | Yes | … | … | … | … | … |
|  | No | … | … | … | … | … |
| Glyceric acid | Yes | … | … | … | … | … |
|  | No | … | … | … | … | … |
| Glycine | Yes | 4 | −0.44 (−1.11 to 0.23) | 0.197 | 83.4% | <0.001 |
|  | No | 3 | −2.75 (−5.36 to −0.15) | 0.038 | 98.3% | <0.001 |
| Glycoursodeoxycholic acid | Yes | … | … | … | … | … |
|  | No | … | … | … | … | … |
| Homovanillic acid | Yes | … | … | … | … | … |
|  | No | … | … | … | … | … |
| Hydroxykynurenine | Yes | 5 | 0.08 (−0.16 to 0.33) | 0.516 | 35.9% | 0.182 |
|  | No | … | … | … | … | … |
| Hypoxanthine | Yes | 3 | −0.80 (−1.90 to 0.30) | 0.156 | 89.8% | <0.001 |
|  | No | 3 | −0.54 (−0.97 to −0.11) | 0.014 | 68.6% | 0.041 |
| Indoleacetic acid | Yes | … | … | … | … | … |
|  | No | … | … | … | … | … |
| Isocitric acid | Yes | … | … | … | … | … |
|  | No | … | … | … | … | … |
| Kynurenic acid | Yes | 8 | −0.39 (−0.60 to −0.17) | 0.001 | 31.4% | 0.177 |
|  | No | 5 | −0.45 (−0.59 to −0.32) | <0.001 | 0.0% | 0.875 |
| L-Acetylcarnitine | Yes | … | … | … | … | … |
|  | No | … | … | … | … | … |
| L-Alanine | Yes | 4 | −0.13 (−0.68 to 0.41) | 0.633 | 75.6% | 0.006 |
|  | No | 3 | −2.28 (−4.81 to 0.26) | 0.078 | 98.3% | <0.001 |
| L-alpha-Aminobutyric acid | Yes | … | … | … | … | … |
|  | No | … | … | … | … | … |
| L-Arginine | Yes | 4 | 0.36 (−0.59 to 1.32) | 0.454 | 90.6% | <0.001 |
|  | No | 5 | −0.31 (−1.31 to 0.70) | 0.552 | 96.4% | <0.001 |
| L-Asparagine | Yes | 3 | −0.43 (−1.36 to 0.50) | 0.366 | 85.5% | 0.001 |
|  | No | 3 | −2.46 (−4.85 to −0.08) | 0.043 | 98.1% | <0.001 |
| L-Aspartic acid | Yes | 3 | 0.43 (−0.99 to 1.84) | 0.554 | 93.4% | <0.001 |
|  | No | 3 | −0.37 (−1.04 to 0.30) | 0.280 | 84.3% | 0.002 |
| L-Carnitine | Yes | … | … | … | … | … |
|  | No | … | … | … | … | … |
| L-Cystine | Yes | … | … | … | … | … |
|  | No | … | … | … | … | … |
| L-Glutamic acid | Yes | 4 | 0.45 (−0.45 to 1.36) | 0.327 | 90.6% | <0.001 |
|  | No | 3 | −0.45 (−2.05 to 1.14) | 0.578 | 96.9% | <0.001 |
| L-Glutamine | Yes | 5 | −0.61 (−1.30 to 0.07) | 0.080 | 88.5% | <0.001 |
|  | No | 3 | −3.31 (−6.00 to −0.62) | 0.016 | 98.3% | <0.001 |
| L-Histidine | Yes | 4 | −0.28 (−0.54 to −0.03) | 0.031 | 0.0% | 0.944 |
|  | No | 3 | 0.64 (−0.79 to 2.07) | 0.381 | 96.2% | <0.001 |
| Linoleic acid | Yes | … | … | … | … | … |
|  | No | … | … | … | … | … |
| L-Isoleucine | Yes | 4 | −0.12 (−0.52 to 0.28) | 0.569 | 55.6% | 0.080 |
|  | No | 3 | −1.35 (−3.56 to 0.87) | 0.233 | 98.0% | <0.001 |
| L-Kynurenine | Yes | 10 | −0.35 (−0.61 to −0.09) | 0.009 | 66.7% | 0.001 |
|  | No | 8 | 0.03 (−0.26 to 0.31) | 0.864 | 89.0% | <0.001 |
| L-Lactic acid | Yes | 3 | −0.13 (−0.59 to 0.32) | 0.564 | 54.4% | 0.112 |
|  | No | … | … | … | … | … |
| L-Leucine | Yes | 4 | −0.38 (−0.70 to −0.07) | 0.016 | 28.4% | 0.242 |
|  | No | 3 | 0.10 (−0.15 to 0.35) | 0.450 | 0.0% | 0.868 |
| L-Lysine | Yes | 3 | −0.24 (−1.03 to 0.54) | 0.545 | 80.2% | 0.006 |
|  | No | 3 | −1.94 (−4.21 to 0.33) | 0.094 | 98.0% | <0.001 |
| L-Malic acid | Yes | … | … | … | … | … |
|  | No | … | … | … | … | … |
| L-Methionine | Yes | 4 | −0.43 (−0.97 to 0.11) | 0.123 | 75.7% | 0.006 |
|  | No | 4 | −1.16 (−2.47 to 0.15) | 0.083 | 96.8% | <0.001 |
| L-Phenylalanine | Yes | 4 | 0.08 (−0.33 to 0.50) | 0.687 | 57.9% | 0.068 |
|  | No | 3 | 1.57 (−0.36 to 3.51) | 0.111 | 97.5% | <0.001 |
| L-Proline | Yes | 3 | 0.34 (0.01 to 0.67) | 0.044 | 0.0% | 0.639 |
|  | No | … | … | … | … | … |
| L-Serine | Yes | 4 | −0.25 (−0.67 to 0.17) | 0.236 | 51.9% | 0.101 |
|  | No | 3 | −2.86 (−5.34 to −0.38) | 0.024 | 98.1% | <0.001 |
| L-Threonine | Yes | 4 | −0.20 (−0.48 to 0.08) | 0.167 | 0.0% | 0.417 |
|  | No | 3 | −1.81 (−3.86 to 0.24) | 0.084 | 97.6% | <0.001 |
| L-Tryptophan | Yes | 13 | −0.41 (−0.60 to −0.22) | <0.001 | 49.8% | 0.021 |
|  | No | 12 | −0.59 (−0.92 to −0.26) | <0.001 | 93.3% | <0.001 |
| L-Tyrosine | Yes | 5 | −0.33 (−0.68 to 0.01) | 0.060 | 58.5% | 0.047 |
|  | No | 3 | −0.33 (−1.24 to 0.57) | 0.471 | 91.4% | <0.001 |
| L-Valine | Yes | 4 | −0.19 (−0.62 to 0.24) | 0.383 | 61.0% | 0.053 |
|  | No | 3 | −2.13 (−4.62 to 0.36) | 0.094 | 98.2% | <0.001 |
| Myo-inositol | Yes | … | … | … | … | … |
|  | No | … | … | … | … | … |
| Oleic acid | Yes | … | … | … | … | … |
|  | No | … | … | … | … | … |
| O-Phosphoethanolamine | Yes | … | … | … | … | … |
|  | No | … | … | … | … | … |
| Ornithine | Yes | 4 | 0.09 (−0.23 to 0.40) | 0.580 | 30.1% | 0.232 |
|  | No | 4 | −0.64 (−1.50 to 0.22) | 0.144 | 94.7% | <0.001 |
| Palmitic acid | Yes | … | … | … | … | … |
|  | No | … | … | … | … | … |
| Palmitoleic acid | Yes | … | … | … | … | … |
|  | No | … | … | … | … | … |
| Phosphatidylcholine (32:0) | Yes | … | … | … | … | … |
|  | No | … | … | … | … | … |
| Phosphatidylcholine (32:1) | Yes | … | … | … | … | … |
|  | No | … | … | … | … | … |
| Phosphatidylethanolamine (34:2) | Yes | … | … | … | … | … |
|  | No | … | … | … | … | … |
| Pyroglutamic acid | Yes | … | … | … | … | … |
|  | No | … | … | … | … | … |
| Pyruvic acid | Yes | … | … | … | … | … |
|  | No | … | … | … | … | … |
| Quinolinic acid | Yes | 7 | −0.08 (−0.46 to 0.30) | 0.678 | 75.8% | <0.001 |
|  | No | 3 | −0.04 (−0.39 to 0.31) | 0.821 | 58.7% | 0.089 |
| Sarcosine | Yes | … | … | … | … | … |
|  | No | … | … | … | … | … |
| Serotonin | Yes | 4 | −0.01 (−0.26 to 0.24) | 0.944 | 0.0% | 0.859 |
|  | No | … | … | … | … | … |
| Stearic acid | Yes | … | … | … | … | … |
|  | No | … | … | … | … | … |
| Succinic acid | Yes | … | … | … | … | … |
|  | No | … | … | … | … | … |
| Symmetric dimethylarginine | Yes | … | … | … | … | … |
|  | No | … | … | … | … | … |
| Taurine | Yes | 3 | −0.32 (−0.59 to −0.05) | 0.021 | 0.0% | 0.561 |
|  | No | 3 | −0.35 (−1.02 to 0.32) | 0.303 | 84.7% | 0.001 |
| Taurochenodesoxycholic acid | Yes | … | … | … | … | … |
|  | No | … | … | … | … | … |
| Tyramine | Yes | … | … | … | … | … |
|  | No | … | … | … | … | … |
| Urea | Yes | … | … | … | … | … |
|  | No | … | … | … | … | … |
| *CI* confidence interval, *SMD* standardized mean differences  ^a^ *p-*Value for between-group effect sizes  ^b^ *p-*Value for heterogeneity calculated using a chi-square analysis | | | | | | |
