# Supplementary material for: An integrated meta-analysis of peripheral blood metabolites and biological functions in major depressive disorder
Source: Mol Psychiatry. 2020 Jan 20;26(8):4265–76. doi: 10.1038/s41380-020-0645-4 (PMC8550972; doi:10.1038/s41380-020-0645-4)
Supplement: Supplementary file 5 — Supplementary Table 4 [file 41380_2020_645_MOESM5_ESM.docx]

| **Supplementary Table 4** Sensitivity analyses, according to mean age | | | | | | |
| --- | --- | --- | --- | --- | --- | --- |
| **Metabolites** | **Mean age** | **No. of comparisons** | **SMD (95% CI)** | ***p-*Value^a^**  **(overall)** | ***I*^2^** | ***p-*Value^b^**  **(heterogeneity)** |
| 1-Methylhistidine | >18 | 4 | −0.33 (−1.01 to 0.35) | 0.342 | 85.7% | <0.001 |
| 25-Hydroxyvitamin D | >18 | … | … | … | … | … |
| 2-Hydroxybutyric acid | >18 | 3 | 1.02 (0.69 to 1.36) | <0.001 | 0.0% | 0.652 |
| 3-Aminoisobutanoic acid | >18 | 4 | −0.06 (−1.03 to 0.92) | 0.911 | 93.0% | <0.001 |
| 3-Hydroxybutyric acid | >18 | 4 | 0.11 (−0.41 to 0.63) | 0.670 | 74.6% | 0.008 |
| 4-Hydroxyproline | >18 | 5 | −0.16 (−0.97 to 0.65) | 0.701 | 90.3% | <0.001 |
| 5-Hydroxylysine | >18 | 3 | 0.73 (−1.22 to 2.68) | 0.464 | 97.3% | <0.001 |
| Adenosine diphosphate | >18 | 3 | 2.01 (−1.20 to 5.22) | 0.220 | 98.0% | <0.001 |
| Aminoadipic acid | >18 | 5 | 1.15 (−0.22 to 2.51) | 0.100 | 96.4% | <0.001 |
| Arachidonic acid | >18 | … | … | … | … | … |
| Asymmetric dimethylarginine | >18 | 4 | 2.52 (0.68 to 4.36) | 0.007 | 97.9% | <0.001 |
| beta-Alanine | >18 | 5 | −0.02 (−0.47 to 0.43) | 0.944 | 70.5% | 0.009 |
| Betaine | >18 | 3 | 0.75 (−0.28 to 1.78) | 0.155 | 88.6% | <0.001 |
| Capric acid | >18 | … | … | … | … | … |
| Cholesterol | >18 | 3 | 0.00 (−1.17 to 1.16) | 0.997 | 92.3% | <0.001 |
| Choline | >18 | 4 | 0.75 (−0.72 to 2.22) | 0.316 | 96.2% | <0.001 |
| cis-Aconitic acid | >18 | 3 | 1.42 (−1.50 to 4.35) | 0.341 | 97.9% | <0.001 |
| Citric acid | >18 | 5 | −0.96 (−1.97 to 0.05) | 0.063 | 92.5% | <0.001 |
| Citrulline | >18 | 8 | 0.18 (−0.31 to 0.68) | 0.466 | 89.3% | <0.001 |
| Creatine | >18 | 4 | −0.80 (−2.05 to 0.46) | 0.213 | 94.9% | <0.001 |
| Creatinine | >18 | 4 | −1.97 (−3.72 to −0.22) | 0.027 | 96.7% | <0.001 |
| Deoxycholic acid | >18 | 3 | −0.17 (−0.60 to 0.25) | 0.427 | 61.1% | 0.076 |
| Dimethylglycine | >18 | 3 | 1.18 (−1.08 to 3.44) | 0.305 | 97.0% | <0.001 |
| Dodecanoic acid | >18 | … | … | … | … | … |
| Ethanolamine | >18 | 5 | −0.94 (−2.84 to 0.97) | 0.336 | 98.1% | <0.001 |
| Gamma-Aminobutyric acid | >18 | 7 | 0.81 (−0.17 to 1.79) | 0.104 | 94.5% | <0.001 |
| Gluconic acid | >18 | 3 | −0.27 (−0.58 to 0.05) | 0.099 | 0.0% | 0.793 |
| Glyceric acid | >18 | 4 | 1.29 (−0.76 to 3.35) | 0.217 | 97.1% | <0.001 |
| Glycine | >18 | 8 | −0.91 (−1.82 to 0.01) | 0.051 | 95.7% | <0.001 |
| Glycoursodeoxycholic acid | >18 | 3 | 0.19 (−0.19 to 0.58) | 0.327 | 53.5% | 0.116 |
| Homovanillic acid | >18 | 3 | 1.36 (−0.80 to 3.52) | 0.216 | 97.8% | <0.001 |
| Hydroxykynurenine | >18 | 6 | 0.03 (−0.19 to 0.25) | 0.804 | 37.7% | 0.155 |
| Hypoxanthine | >18 | 4 | −0.46 (−0.98 to 0.05) | 0.076 | 78.8% | 0.003 |
| Indoleacetic acid | >18 | 3 | 1.49 (−1.03 to 4.00) | 0.247 | 97.6% | <0.001 |
| Isocitric acid | >18 | 4 | 1.06 (−0.98 to 3.10) | 0.308 | 97.4% | <0.001 |
| Kynurenic acid | >18 | 12 | −0.41 (−0.53 to −0.30) | <0.001 | 9.0% | 0.357 |
| L-Acetylcarnitine | >18 | 3 | −2.06 (−3.58 to −0.54) | 0.008 | 95.4% | <0.001 |
| L-Alanine | >18 | 8 | −0.67 (−1.48 to 0.15) | 0.109 | 94.8% | <0.001 |
| L-alpha-Aminobutyric acid | >18 | 3 | 0.17 (−0.40 to 0.73) | 0.563 | 77.4% | 0.012 |
| L-Arginine | >18 | 7 | −0.51 (−1.11 to 0.09) | 0.097 | 91.9% | <0.001 |
| L-Asparagine | >18 | 6 | −1.33 (−2.45 to −0.21) | 0.020 | 95.7% | <0.001 |
| L-Aspartic acid | >18 | 7 | 0.09 (−0.53 to 0.72) | 0.771 | 90.0% | <0.001 |
| L-Carnitine | >18 | 3 | −0.40 (−1.18 to 0.37) | 0.308 | 88.2% | <0.001 |
| L-Cystine | >18 | 3 | 0.25 (−2.70 to 3.20) | 0.870 | 98.5% | <0.001 |
| L-Glutamic acid | >18 | 7 | 0.06 (−0.73 to 0.86) | 0.873 | 94.1% | <0.001 |
| L-Glutamine | >18 | 8 | −1.28 (−2.19 to −0.37) | 0.006 | 95.8% | <0.001 |
| L-Histidine | >18 | 7 | 0.09 (−0.51 to 0.70) | 0.763 | 90.2% | <0.001 |
| Linoleic acid | >18 | 3 | −0.77 (−1.66 to 0.12) | 0.089 | 76.0% | 0.016 |
| L-Isoleucine | >18 | 7 | −0.58 (−1.40 to 0.25) | 0.171 | 94.4% | <0.001 |
| L-Kynurenine | >18 | 18 | −0.17 (−0.36 to 0.02) | 0.077 | 81.9% | <0.001 |
| L-Lactic acid | >18 | 5 | −0.37 (−1.23 to 0.49) | 0.400 | 92.3% | <0.001 |
| L-Leucine | >18 | 8 | −0.14 (−0.37 to 0.08) | 0.217 | 41.5% | 0.102 |
| L-Lysine | >18 | 6 | −1.03 (−2.10 to 0.04) | 0.059 | 95.4% | <0.001 |
| L-Malic acid | >18 | 3 | 0.87 (−0.57 to 2.31) | 0.235 | 93.7% | <0.001 |
| L-Methionine | >18 | 8 | −0.73 (−1.40 to −0.06) | 0.032 | 93.4% | <0.001 |
| L-Phenylalanine | >18 | 7 | 0.68 (−0.08 to 1.44) | 0.079 | 93.4% | <0.001 |
| L-Proline | >18 | 6 | −0.35 (−1.40 to 0.70) | 0.510 | 94.9% | <0.001 |
| L-Serine | >18 | 8 | −0.90 (−1.73 to −0.07) | 0.034 | 94.5% | <0.001 |
| L-Threonine | >18 | 8 | −0.64 (−1.36 to 0.09) | 0.085 | 93.1% | <0.001 |
| L-Tryptophan | >18 | 26 | −0.46 (−0.66 to −0.26) | <0.001 | 87.8% | <0.001 |
| L-Tyrosine | >18 | 8 | −0.31 (−0.69 to 0.06) | 0.099 | 79.4% | <0.001 |
| L-Valine | >18 | 8 | −0.71 (−1.48 to 0.06) | 0.071 | 94.3% | <0.001 |
| Myo-inositol | >18 | 3 | −0.79 (−1.17 to −0.41) | <0.001 | 28.3% | 0.248 |
| Oleic acid | >18 | 3 | −0.53 (−1.30 to 0.25) | 0.183 | 69.4% | 0.038 |
| O-Phosphoethanolamine | >18 | 4 | −0.21 (−0.75 to 0.33) | 0.448 | 78.3% | 0.003 |
| Ornithine | >18 | 8 | −0.31 (−0.78 to 0.17) | 0.205 | 89.1% | <0.001 |
| Palmitic acid | >18 | … | … | … | … | … |
| Palmitoleic acid | >18 | … | … | … | … | … |
| Phosphatidylcholine (32:0) | >18 | 3 | 0.08 (−0.51 to 0.66) | 0.799 | 80.6% | 0.006 |
| Phosphatidylcholine (32:1) | >18 | 3 | 0.56 (0.32 to 0.80) | <0.001 | 0.0% | 0.482 |
| Phosphatidylethanolamine (34:2) | >18 | 3 | 0.38 (−0.12 to 0.87) | 0.136 | 73.0% | 0.025 |
| Pyroglutamic acid | >18 | 4 | −0.54 (−2.03 to 0.95) | 0.476 | 95.8% | <0.001 |
| Pyruvic acid | >18 | 3 | −0.92 (−1.68 to −0.17) | 0.017 | 80.7% | 0.006 |
| Quinolinic acid | >18 | 9 | −0.03 (−0.30 to 0.24) | 0.822 | 71.8% | <0.001 |
| Sarcosine | >18 | 4 | −0.20 (−0.95 to 0.55) | 0.602 | 86.5% | <0.001 |
| Serotonin | >18 | 5 | −0.26 (−0.76 to 0.23) | 0.301 | 77.9% | 0.001 |
| Stearic acid | >18 | 3 | −0.42 (−1.00 to 0.16) | 0.152 | 47.7% | 0.148 |
| Succinic acid | >18 | 4 | 0.69 (−0.35 to 1.74) | 0.194 | 94.4% | <0.001 |
| Symmetric dimethylarginine | >18 | 3 | 1.05 (−0.75 to 2.84) | 0.253 | 97.7% | <0.001 |
| Taurine | >18 | 6 | −0.33 (−0.65 to −0.01) | 0.043 | 65.0% | 0.014 |
| Taurochenodesoxycholic acid | >18 | 3 | 0.28 (0.04 to 0.52) | 0.024 | 0.5% | 0.366 |
| Tyramine | >18 | 3 | 1.12 (0.70 to 1.55) | <0.001 | 58.8% | 0.089 |
| Urea | >18 | 3 | −2.11 (−4.59 to 0.38) | 0.097 | 97.9% | <0.001 |
| *CI* confidence interval, *SMD* standardized mean differences  ^a^ *p-*Value for between-group effect sizes  ^b^ *p-*Value for heterogeneity calculated using a chi-square analysis | | | | | | |
