# Supplementary material for: An integrated meta-analysis of peripheral blood metabolites and biological functions in major depressive disorder
Source: Mol Psychiatry. 2020 Jan 20;26(8):4265–76. doi: 10.1038/s41380-020-0645-4 (PMC8550972; doi:10.1038/s41380-020-0645-4)
Supplement: Supplementary file 6 — Supplementary Table 5 [file 41380_2020_645_MOESM6_ESM.docx]

| **Supplementary Table 5** Sensitivity analyses, according to analytic technique | | | | | | |
| --- | --- | --- | --- | --- | --- | --- |
| **Metabolites** | **Analytic technique** | **No. of comparisons** | **SMD (95% CI)** | ***p-*Value^a^**  **(overall)** | ***I*^2^** | ***p-*Value^b^**  **(heterogeneity)** |
| 1-Methylhistidine | MS | 4 | −0.33 (−1.01 to 0.35) | 0.342 | 85.7% | <0.001 |
| 25-Hydroxyvitamin D | MS | 3 | −0.29 (−0.39 to −0.18) | <0.001 | 0.0% | 0.811 |
| 2-Hydroxybutyric acid | MS | 3 | 1.02 (0.69 to 1.36) | <0.001 | 0.0% | 0.652 |
| 3-Aminoisobutanoic acid | MS | 4 | −0.06 (−1.03 to 0.92) | 0.911 | 93.0% | <0.001 |
| 3-Hydroxybutyric acid | MS | 3 | 0.02 (−0.67 to 0.72) | 0.952 | 76.8% | 0.013 |
| 4-Hydroxyproline | MS | 5 | −0.16 (−0.97 to 0.65) | 0.701 | 90.3% | <0.001 |
| 5-Hydroxylysine | MS | 3 | 0.73 (−1.22 to 2.68) | 0.464 | 97.3% | <0.001 |
| Adenosine diphosphate | MS | 3 | 2.01 (−1.20 to 5.22) | 0.220 | 98.0% | <0.001 |
| Aminoadipic acid | MS | 5 | 1.15 (−0.22 to 2.51) | 0.100 | 96.4% | <0.001 |
| Arachidonic acid | MS | 3 | −0.52 (−1.42 to 0.37) | 0.253 | 84.4% | 0.002 |
| Asymmetric dimethylarginine | MS | 4 | 2.52 (0.68 to 4.36) | 0.007 | 97.9% | <0.001 |
| beta-Alanine | MS | 5 | −0.02 (−0.47 to 0.43) | 0.944 | 70.5% | 0.009 |
| Betaine | MS | 4 | 0.26 (−0.99 to 1.51) | 0.679 | 95.3% | <0.001 |
| Capric acid | MS | 4 | −1.31 (−2.65 to 0.03) | 0.056 | 95.6% | <0.001 |
| Cholesterol | MS | … | … | … | … | … |
| Choline | MS | 3 | 1.18 (−0.94 to 3.30) | 0.276 | 96.6% | <0.001 |
| cis-Aconitic acid | MS | 3 | 1.42 (−1.50 to 4.35) | 0.341 | 97.9% | <0.001 |
| Citric acid | MS | 4 | −1.03 (−2.54 to 0.48) | 0.181 | 94.4% | <0.001 |
| Citrulline | MS | 8 | 0.18 (−0.31 to 0.68) | 0.466 | 89.3% | <0.001 |
| Creatine | MS | 4 | −0.87 (−2.09 to 0.35) | 0.163 | 94.7% | <0.001 |
| Creatinine | MS | 4 | −2.10 (−3.87 to −0.33) | 0.020 | 96.7% | <0.001 |
| Deoxycholic acid | MS | 3 | −0.17 (−0.60 to 0.25) | 0.427 | 61.1% | 0.076 |
| Dimethylglycine | MS | 3 | 1.18 (−1.08 to 3.44) | 0.305 | 97.0% | <0.001 |
| Dodecanoic acid | MS | 4 | −0.73 (−0.99 to −0.48) | <0.001 | 0.0% | 0.544 |
| Ethanolamine | MS | 5 | −0.94 (−2.84 to 0.97) | 0.336 | 98.1% | <0.001 |
| Gamma-Aminobutyric acid | MS | 7 | 0.81 (−0.17 to 1.79) | 0.104 | 94.5% | <0.001 |
| Gluconic acid | MS | 3 | −0.27 (−0.58 to 0.05) | 0.099 | 0.0% | 0.793 |
| Glyceric acid | MS | 4 | 1.29 (−0.76 to 3.35) | 0.217 | 97.1% | <0.001 |
| Glycine | MS | 7 | −0.87 (−1.88 to 0.13) | 0.088 | 95.6% | <0.001 |
| Glycoursodeoxycholic acid | MS | 3 | 0.19 (−0.19 to 0.58) | 0.327 | 53.5% | 0.116 |
| Homovanillic acid | MS | 3 | 1.36 (−0.80 to 3.52) | 0.216 | 97.8% | <0.001 |
| Hydroxykynurenine | MS | 6 | 0.03 (−0.19 to 0.25) | 0.804 | 37.7% | 0.155 |
| Hypoxanthine | MS | 6 | −0.70 (−1.21 to −0.18) | 0.009 | 85.9% | <0.001 |
| Indoleacetic acid | MS | 3 | 1.49 (−1.03 to 4.00) | 0.247 | 97.6% | <0.001 |
| Isocitric acid | MS | 4 | 1.06 (−0.98 to 3.10) | 0.308 | 97.4% | <0.001 |
| Kynurenic acid | MS | 13 | −0.42 (−0.52 to −0.31) | <0.001 | 1.2% | 0.434 |
| L-Acetylcarnitine | MS | 3 | −2.06 (−3.58 to −0.54) | 0.008 | 95.4% | <0.001 |
| L-Alanine | MS | 7 | −0.70 (−1.64 to 0.25) | 0.148 | 95.1% | <0.001 |
| L-alpha-Aminobutyric acid | MS | 3 | 0.17 (−0.40 to 0.73) | 0.563 | 77.4% | 0.012 |
| L-Arginine | MS | 9 | −0.01 (−0.73 to 0.70) | 0.970 | 95.2% | <0.001 |
| L-Asparagine | MS | 6 | −1.33 (−2.45 to −0.21) | 0.020 | 95.7% | <0.001 |
| L-Aspartic acid | MS | 7 | 0.09 (−0.53 to 0.72) | 0.771 | 90.0% | <0.001 |
| L-Carnitine | MS | 3 | −0.40 (−1.18 to 0.37) | 0.308 | 88.2% | <0.001 |
| L-Cystine | MS | 3 | 0.25 (−2.70 to 3.20) | 0.870 | 98.5% | <0.001 |
| L-Glutamic acid | MS | 7 | 0.06 (−0.73 to 0.86) | 0.873 | 94.1% | <0.001 |
| L-Glutamine | MS | 7 | −1.46 (−2.55 to −0.36) | 0.009 | 96.4% | <0.001 |
| L-Histidine | MS | 6 | 0.15 (−0.60 to 0.89) | 0.697 | 91.7% | <0.001 |
| Linoleic acid | MS | 5 | −0.99 (−1.46 to −0.51) | <0.001 | 63.4% | 0.027 |
| L-Isoleucine | MS | 6 | −0.60 (−1.61 to 0.41) | 0.242 | 95.1% | <0.001 |
| L-Kynurenine | MS | 18 | −0.17 (−0.36 to 0.02) | 0.077 | 81.9% | <0.001 |
| L-Lactic acid | MS | 4 | −0.34 (−1.53 to 0.85) | 0.571 | 94.1% | <0.001 |
| L-Leucine | MS | 7 | −0.04 (−0.22 to 0.15) | 0.682 | 0.0% | 0.452 |
| L-Lysine | MS | 6 | −1.03 (−2.10 to 0.04) | 0.059 | 95.4% | <0.001 |
| L-Malic acid | MS | 3 | 0.87 (−0.57 to 2.31) | 0.235 | 93.7% | <0.001 |
| L-Methionine | MS | 8 | −0.73 (−1.40 to −0.06) | 0.032 | 93.4% | <0.001 |
| L-Phenylalanine | MS | 7 | 0.68 (−0.08 to 1.44) | 0.079 | 93.4% | <0.001 |
| L-Proline | MS | 6 | −0.35 (−1.40 to 0.70) | 0.510 | 94.9% | <0.001 |
| L-Serine | MS | 8 | −0.90 (−1.73 to −0.07) | 0.034 | 94.5% | <0.001 |
| L-Threonine | MS | 8 | −0.64 (−1.36 to 0.09) | 0.085 | 93.1% | <0.001 |
| L-Tryptophan | MS | 26 | −0.46 (−0.66 to −0.26) | <0.001 | 87.8% | <0.001 |
| L-Tyrosine | MS | 7 | −0.25 (−0.65 to 0.16) | 0.236 | 79.0% | <0.001 |
| L-Valine | MS | 7 | −0.76 (−1.66 to 0.15) | 0.101 | 94.8% | <0.001 |
| Myo-inositol | MS | … | … | … | … | … |
| Oleic acid | MS | 5 | −0.84 (−1.31 to −0.36) | 0.001 | 63.6% | 0.027 |
| O-Phosphoethanolamine | MS | 4 | −0.21 (−0.75 to 0.33) | 0.448 | 78.3% | 0.003 |
| Ornithine | MS | 8 | −0.31 (−0.78 to 0.17) | 0.205 | 89.1% | <0.001 |
| Palmitic acid | MS | 4 | −0.69 (−1.23 to −0.16) | 0.011 | 71.2% | 0.015 |
| Palmitoleic acid | MS | 3 | −0.90 (−1.70 to −0.10) | 0.027 | 79.8% | 0.007 |
| Phosphatidylcholine (32:0) | MS | 3 | 0.08 (−0.51 to 0.66) | 0.799 | 80.6% | 0.006 |
| Phosphatidylcholine (32:1) | MS | 3 | 0.56 (0.32 to 0.80) | <0.001 | 0.0% | 0.482 |
| Phosphatidylethanolamine (34:2) | MS | 3 | 0.38 (−0.12 to 0.87) | 0.136 | 73.0% | 0.025 |
| Pyroglutamic acid | MS | 5 | −0.64 (−1.76 to 0.49) | 0.267 | 95.1% | <0.001 |
| Pyruvic acid | MS | … | … | … | … | … |
| Quinolinic acid | MS | 10 | −0.06 (−0.32 to 0.20) | 0.660 | 69.7% | <0.001 |
| Sarcosine | MS | 4 | −0.20 (−0.95 to 0.55) | 0.602 | 86.5% | <0.001 |
| Serotonin | MS | 5 | −0.26 (−0.76 to 0.23) | 0.301 | 77.9% | 0.001 |
| Stearic acid | MS | 3 | −0.42 (−1.00 to 0.16) | 0.152 | 47.7% | 0.148 |
| Succinic acid | MS | 4 | 0.69 (−0.35 to 1.74) | 0.194 | 94.4% | <0.001 |
| Symmetric dimethylarginine | MS | 3 | 1.05 (−0.75 to 2.84) | 0.253 | 97.7% | <0.001 |
| Taurine | MS | 5 | −0.33 (−0.73 to 0.07) | 0.110 | 71.7% | 0.007 |
| Taurochenodesoxycholic acid | MS | 4 | 0.33 (0.11 to 0.54) | 0.003 | 0.0% | 0.446 |
| Tyramine | MS | 3 | 1.12 (0.70 to 1.55) | <0.001 | 58.8% | 0.089 |
| Urea | MS | 3 | −2.11 (−4.59 to 0.38) | 0.097 | 97.9% | <0.001 |
| *CI* confidence interval, *SMD* standardized mean differences  ^a^ *p-*Value for between-group effect sizes  ^b^ *p-*Value for heterogeneity calculated using a chi-square analysis | | | | | | |
