# Supplementary material for: An integrated meta-analysis of peripheral blood metabolites and biological functions in major depressive disorder
Source: Mol Psychiatry. 2020 Jan 20;26(8):4265–76. doi: 10.1038/s41380-020-0645-4 (PMC8550972; doi:10.1038/s41380-020-0645-4)
Supplement: Supplementary file 7 — Supplementary Table 6 [file 41380_2020_645_MOESM7_ESM.docx]

| **Supplementary Table 6** Meta-regression analyses of metabolites in the blood, comparing MDD patients and controls | | | |
| --- | --- | --- | --- |
| **Variables** | **No. of Comparisons** | **Slope (95% CI)** | ***p-*Value^a^** |
| **Kynurenic acid** |  |  |  |
| Sample size | 13 | 0.000 (−0.001 to 0.001) | 0.985 |
| Proportion of females | 12 | 0.607 (−0.381 to 1.595) | 0.201 |
| Mean age | 12 | 0.002 (−0.007 to 0.011) | 0.665 |
| **L-Kynurenine** |  |  |  |
| Sample size | 18 | 0.000 (−0.001 to 0.001) | 0.972 |
| Proportion of females | 16 | −1.288 (−4.166 to 1.591) | 0.354 |
| Mean age | 16 | 0.000 (−0.044 to 0.044) | 0.993 |
| Disease severity | 11 | −0.013 (−0.056 to 0.031) | 0.525 |
| **L-Tryptophan** |  |  |  |
| Sample size | 26 | 0.000 (0.000 to 0.001) | 0.527 |
| Proportion of females | 23 | 2.097 (−0.759 to 4.952) | 0.142 |
| Mean age | 24 | 0.005 (−0.032 to 0.043) | 0.770 |
| Disease severity | 18 | −0.040 (−0.076 to −0.005) | 0.029 |
| **Quinolinic acid** |  |  |  |
| Sample size | 10 | 0.003 (−0.006 to 0.012) | 0.521 |
| *CI* confidence interval  ^a^ *p-*Value for meta-regression analysis | | | |
