# Supplementary material for: An integrated meta-analysis of peripheral blood metabolites and biological functions in major depressive disorder
Source: Mol Psychiatry. 2020 Jan 20;26(8):4265–76. doi: 10.1038/s41380-020-0645-4 (PMC8550972; doi:10.1038/s41380-020-0645-4)
Supplement: Supplementary file 8 — Supplementary Table 7 [file 41380_2020_645_MOESM8_ESM.docx]

| **Supplemental Table 7** Altered metabolic pathways in the blood of patients with MDD, according to antidepressant exposure | |
| --- | --- |
| **Metabolic pathways** | ***p-*Value^a^** |
| Antidepressant-free major depressive disorder |  |
| Aminoacyl-tRNA biosynthesis | <0.001 |
| Nitrogen metabolism | <0.001 |
| Tryptophan metabolism | 0.002 |
| Synthesis and degradation of ketone bodies | 0.022 |
| Arginine and proline metabolism | 0.031 |
| Antidepressant-treated major depressive disorder |  |
| Aminoacyl-tRNA biosynthesis | <0.001 |
| Nitrogen metabolism | <0.001 |
| Cyanoamino acid metabolism | <0.001 |
| Glycine, serine and threonine metabolism | <0.001 |
| Purine metabolism | 0.002 |
| Alanine, aspartate and glutamate metabolism | 0.002 |
| Methane metabolism | 0.004 |
| Tryptophan metabolism | 0.020 |
| D-Glutamine and D-glutamate metabolism | 0.032 |
| ^a^ *p-*Values were calculated from hypergeometric tests in MetaboAnalyst | |
