# Supplementary material for: An integrated meta-analysis of peripheral blood metabolites and biological functions in major depressive disorder
Source: Mol Psychiatry. 2020 Jan 20;26(8):4265–76. doi: 10.1038/s41380-020-0645-4 (PMC8550972; doi:10.1038/s41380-020-0645-4)
Supplement: Supplementary file 9 — Supplementary Table 8 [file 41380_2020_645_MOESM9_ESM.docx]

| **Supplemental Table 8** Significantly altered canonical pathways in the blood of patients with MDD, according to antidepressant exposure | |
| --- | --- |
| **Canonical pathways** | ***p-*Value^a^** |
| Antidepressant-free major depressive disorder |  |
| tRNA charging | <0.001 |
| Histamine biosynthesis | 0.011 |
| Proline degradation | 0.027 |
| Taurine biosynthesis | 0.032 |
| Antidepressant-treated major depressive disorder |  |
| tRNA charging | <0.001 |
| Glycine biosynthesis I | <0.001 |
| Asparagine biosynthesis I | <0.001 |
| Glutamate receptor signaling | <0.001 |
| dTMP de novo biosynthesis | 0.001 |
| Superpathway of serine and glycine biosynthesis I | 0.001 |
| Glycine betaine degradation | 0.001 |
| Folate polyglutamylation | 0.001 |
| 5-Aminoimidazole ribonucleotide biosynthesis I | 0.002 |
| NAD biosynthesis II (from tryptophan) | 0.003 |
| Folate transformations I | 0.003 |
| Phenylalanine degradation IV (mammalian, via side chain) | 0.003 |
| Purine nucleotides de novo biosynthesis II | 0.006 |
| Bupropion degradation | 0.008 |
| Asparagine degradation I | 0.013 |
| L-Serine degradation | 0.013 |
| Glutamine degradation I | 0.013 |
| Glycine biosynthesis III | 0.017 |
| Phosphatidylethanolamine biosynthesis III | 0.017 |
| Glutamine biosynthesis I | 0.025 |
| Cysteine biosynthesis/homocysteine degradation | 0.025 |
| Glycine biosynthesis II | 0.025 |
| Glycine cleavage complex | 0.029 |
| Glycine degradation (creatine biosynthesis) | 0.029 |
| Threonine degradation II | 0.029 |
| Tetrapyrrole biosynthesis II | 0.033 |
| Glutathione biosynthesis | 0.033 |
| Amyotrophic lateral sclerosis signaling | 0.037 |
| Serine biosynthesis | 0.037 |
| L-Glutamine biosynthesis II (tRNA-dependent) | 0.037 |
| L-Carnitine biosynthesis | 0.042 |
| NAD biosynthesis from 2-amino-3-carboxymuconate semialdehyde | 0.042 |
| Ceramide biosynthesis | 0.042 |
| Tryptophan degradation X (mammalian, via tryptamine) | 0.042 |
| Selenocysteine biosynthesis II (archaea and eukaryotes) | 0.042 |
| Adenine and adenosine salvage III | 0.042 |
| Tryptophan degradation to 2-amino-3-carboxymuconate semialdehyde | 0.042 |
| Serotonin receptor signaling | 0.042 |
| Serotonin and melatonin biosynthesis | 0.046 |
| Leukotriene biosynthesis | 0.046 |
| UDP-N-acetyl-D-glucosamine biosynthesis II | 0.046 |
| Pyrimidine ribonucleotides interconversion | 0.046 |
| Adenosine nucleotides degradation II | 0.046 |
| Glutathione-mediated detoxification | 0.049 |
| Purine ribonucleosides degradation to ribose-1-phosphate | 0.049 |
| ^a^ *p-*Values were calculated from Fisher’s exact tests in Ingenuity Pathway Analysis | |
