# Supplementary figures and images for: An integrated meta-analysis of peripheral blood metabolites and biological functions in major depressive disorder
Source: Mol Psychiatry. 2020 Jan 20;26(8):4265–76. doi: 10.1038/s41380-020-0645-4 (PMC8550972; doi:10.1038/s41380-020-0645-4)

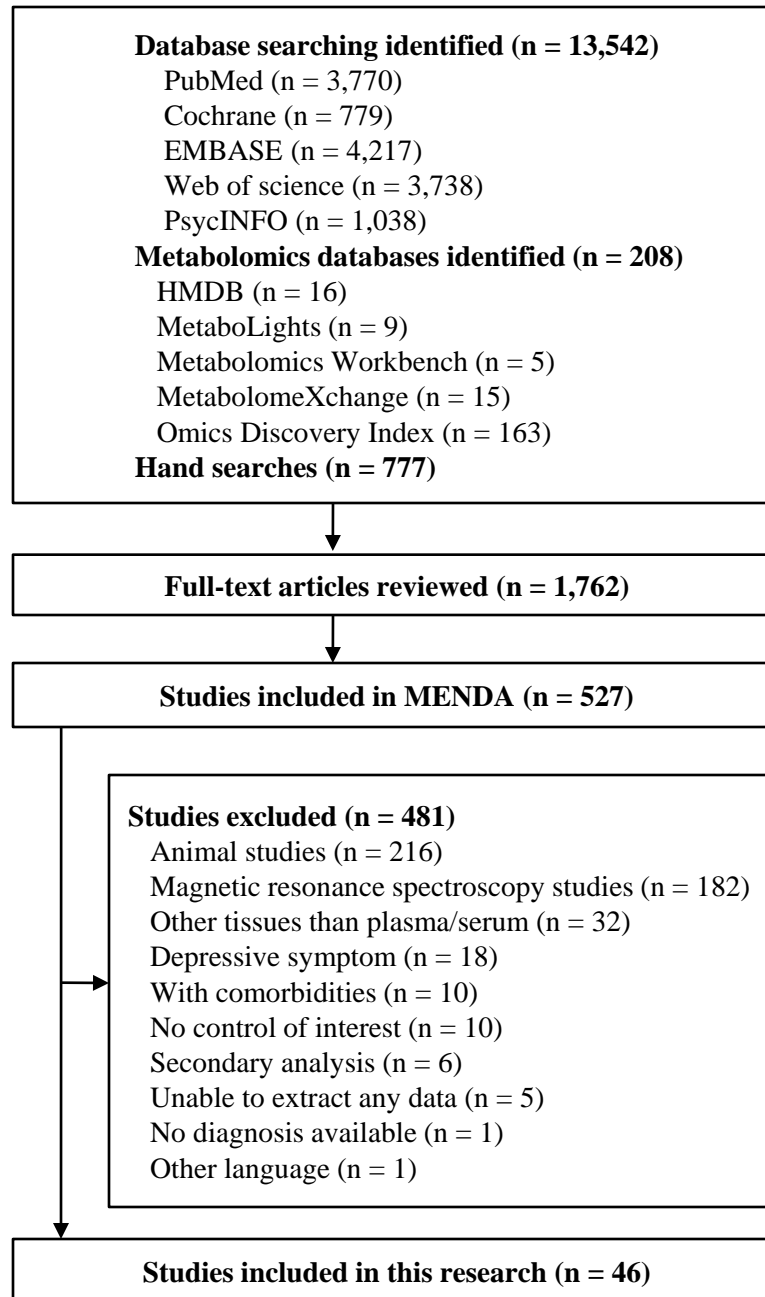

Supplement: Supplementary file 10 — Supplementary Figure 1 [file 41380_2020_645_MOESM10_ESM.pdf]

# Metabolite

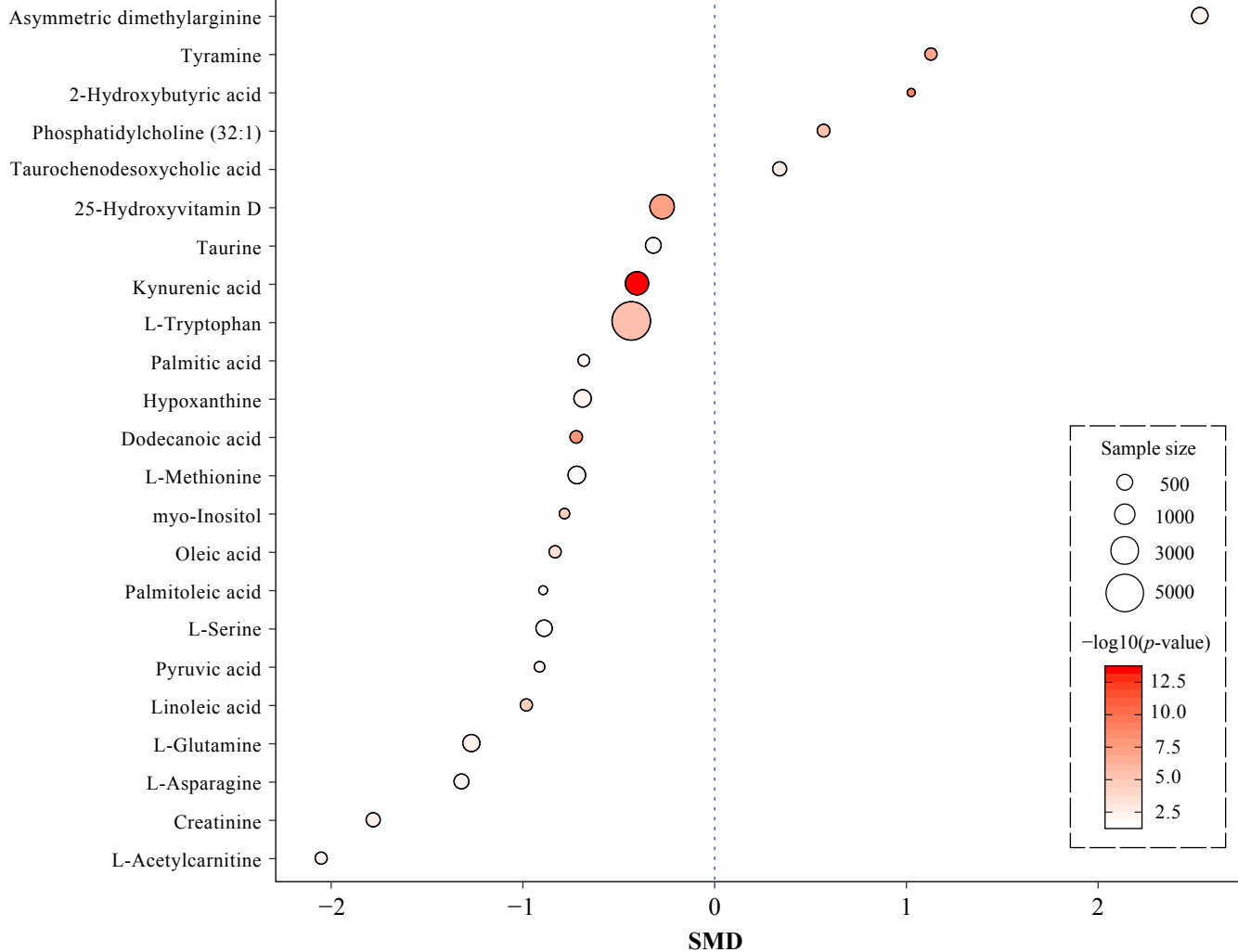

Supplement: Supplementary file 11 — Supplementary Figure 2 [file 41380_2020_645_MOESM11_ESM.pdf]

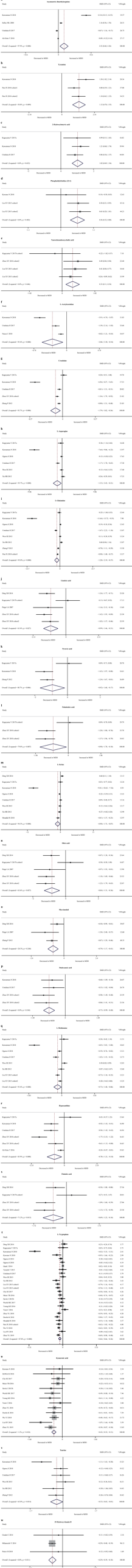

Supplement: Supplementary file 12 — Supplementary Figure 3 [file 41380_2020_645_MOESM12_ESM.pdf]

a

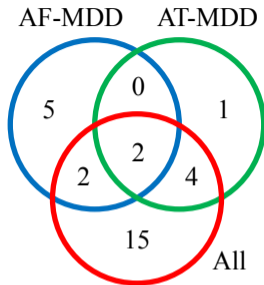

b

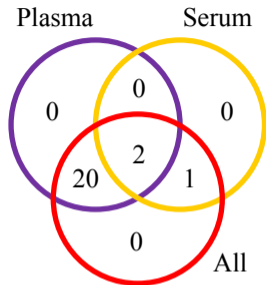

c

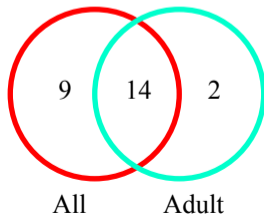

d

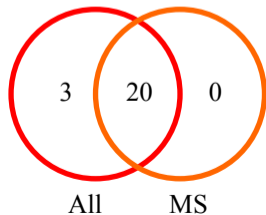

Supplement: Supplementary file 13 — Supplementary Figure 4 [file 41380_2020_645_MOESM13_ESM.pdf]

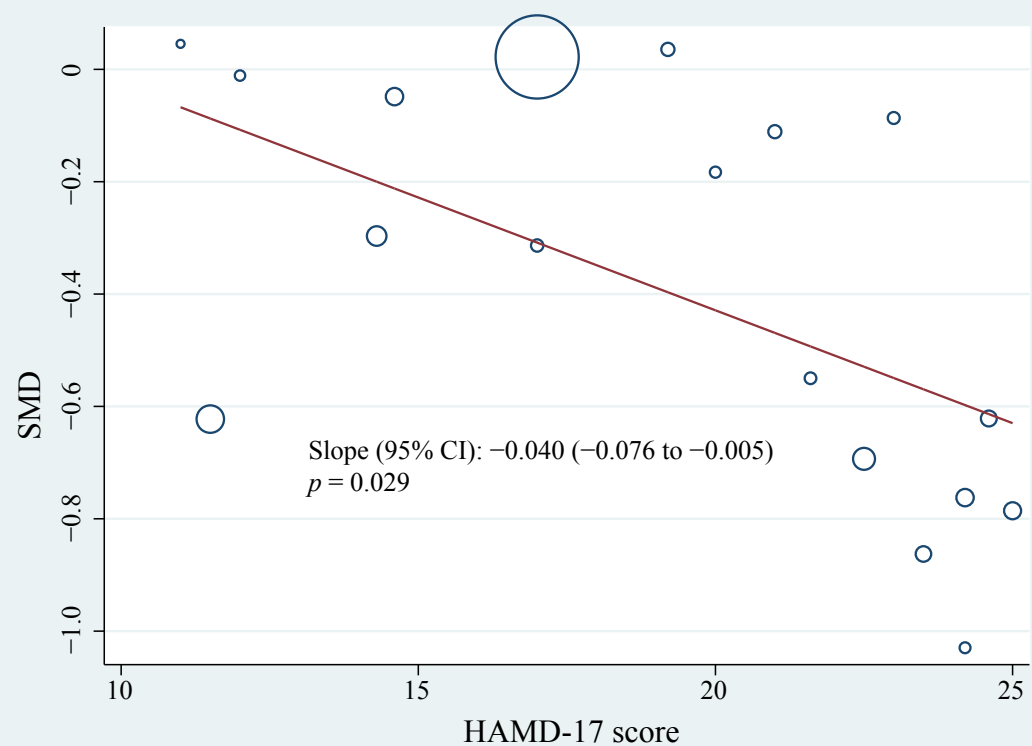

Supplement: Supplementary file 14 — Supplementary Figure 5 [file 41380_2020_645_MOESM14_ESM.pdf]

a

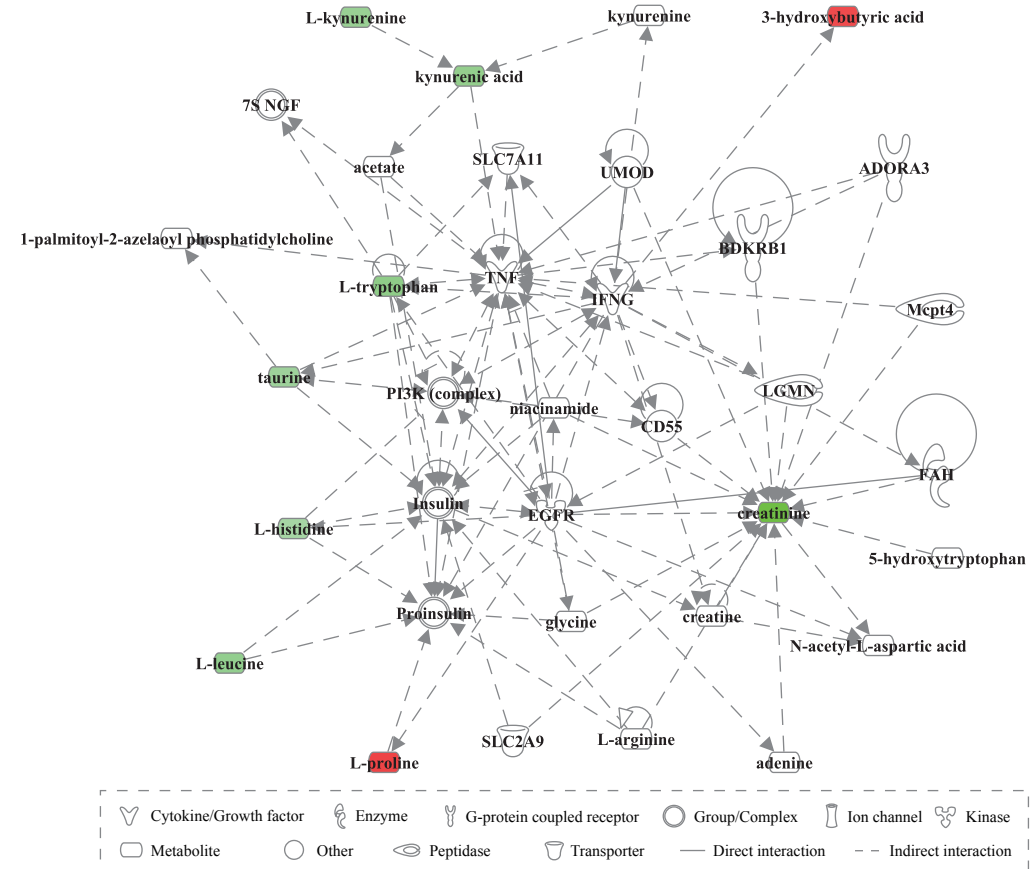

b

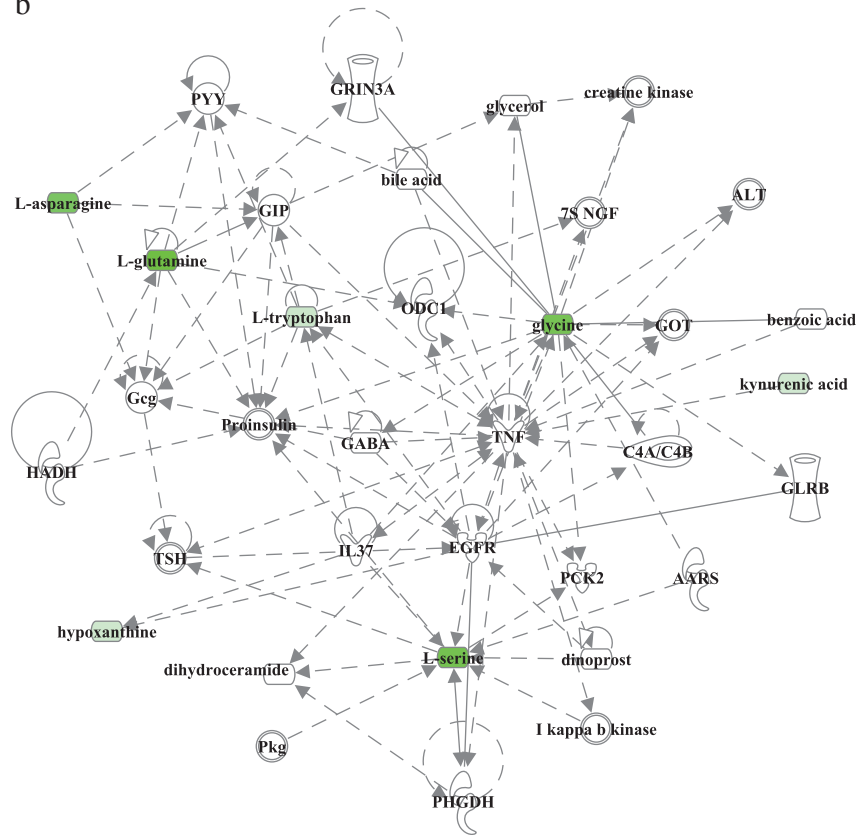

Supplement: Supplementary file 15 — Supplementary Figure 6 [file 41380_2020_645_MOESM15_ESM.pdf]
